# Finding Single Copy Genes Out of Sequenced Genomes for Multilocus Phylogenetics in Non-Model Fungi

**DOI:** 10.1371/journal.pone.0018803

**Published:** 2011-04-13

**Authors:** Nicolas Feau, Thibaut Decourcelle, Claude Husson, Marie-Laure Desprez-Loustau, Cyril Dutech

**Affiliations:** 1 INRA, UMR1202, BIOGECO (Biodiversité Gènes et Communautés), Cestas, France; 2 INRA, Nancy Université, UMR1136 Interactions Arbres/Microorganismes (IFR110), Champenoux, France; University College Dublin, Ireland

## Abstract

Historically, fungal multigene phylogenies have been reconstructed based on a small number of commonly used genes. The availability of complete fungal genomes has given rise to a new wave of model organisms that provide large number of genes potentially useful for building robust gene genealogies. Unfortunately, cross-utilization of these resources to study phylogenetic relationships in the vast majority of non-model fungi (i.e. “orphan” species) remains an unexamined question. To address this problem, we developed a method coupled with a program named “PHYLORPH” (PHYLogenetic markers for ORPHans). The method screens fungal genomic databases (107 fungal genomes fully sequenced) for single copy genes that might be easily transferable and well suited for studies at low taxonomic levels (for example, in species complexes) in non-model fungal species. To maximize the chance to target genes with informative regions, PHYLORPH displays a graphical evaluation system based on the estimation of nucleotide divergence relative to substitution type. The usefulness of this approach was tested by developing markers in four non-model groups of fungal pathogens. For each pathogen considered, 7 to 40% of the 10–15 best candidate genes proposed by PHYLORPH yielded sequencing success. Levels of polymorphism of these genes were compared with those obtained for some genes traditionally used to build fungal phylogenies (e.g. nuclear rDNA, β-tubulin, γ-actin, Elongation factor EF-1α). These genes were ranked among the best-performing ones and resolved accurately taxa relationships in each of the four non-model groups of fungi considered. We envision that PHYLORPH will constitute a useful tool for obtaining new and accurate phylogenetic markers to resolve relationships between closely related non-model fungal species.

## Introduction

Accurate reconstruction of timing and order of species filiations critically depends on obtaining suitable characters for phylogenetic analyses [1]. Transition from single to multi-locus phylogenies has successfully provided novel insights into this field. For example, topological congruence between multigene phylogenies is now commonly used to accurately define phylogenetic species in fungi [2]. Multigene datasets are also useful to address questions in population history, demography and speciation. As theoretically anticipated, different genes can evolve in radically different ways resulting in gene/species trees inconsistencies. Using few genes which contain accurate information can allow the construction of robust species trees [1], [3], [4].

**Figure 1:**
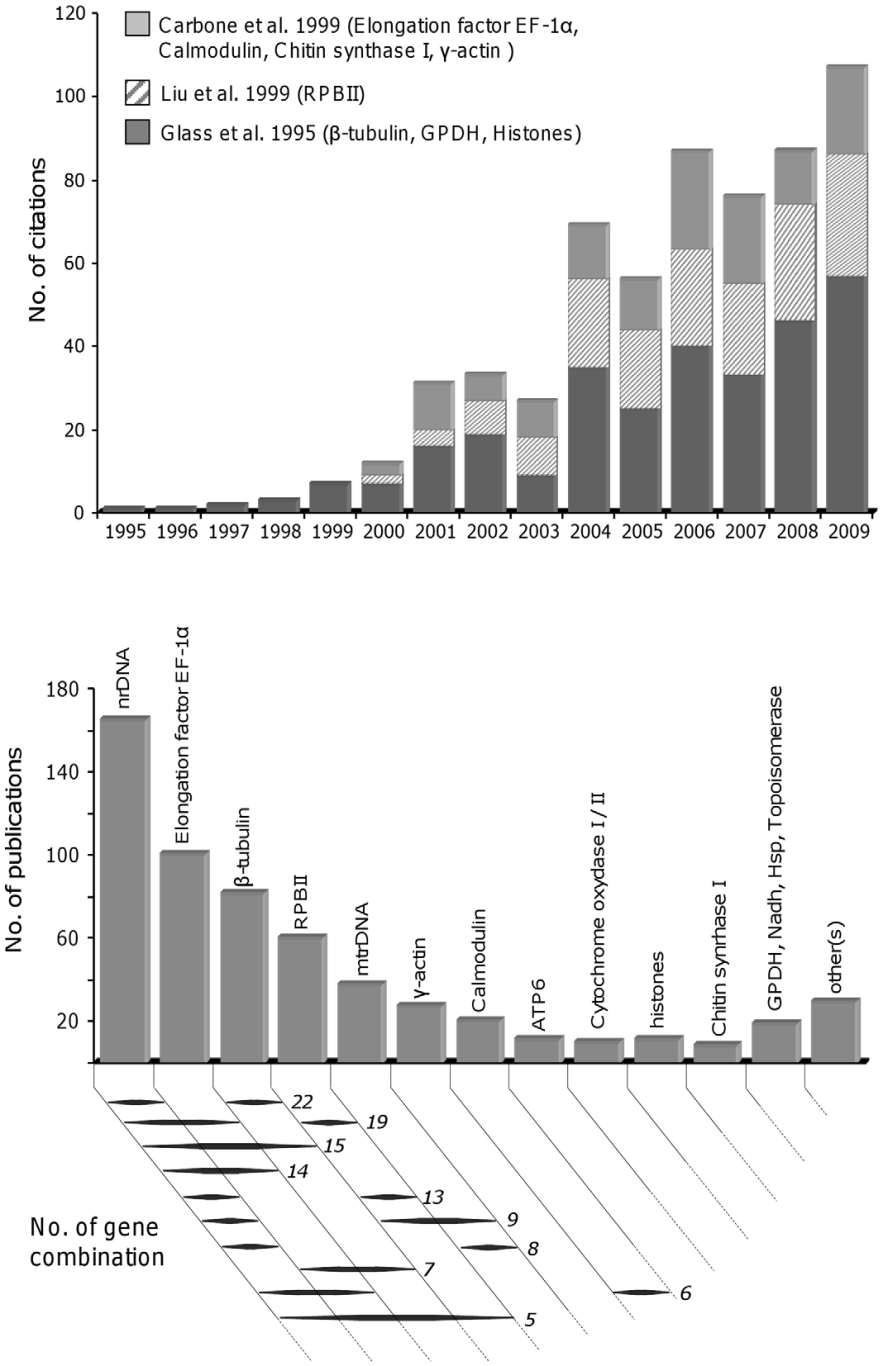
Genes most commonly used in fungal multigene phylogenies. Data were compiled from 196 multigene phylogenies published between 1996 and 2009 retrieved in ISI Thompson Reuters using the following keywords: “fungi” OR “fungal” AND “multigene” or “multilocus” AND “phylogeny”. A, distribution of genes/number of publications; B, occurrence of the ten most commonly gene combination used among the 196 studies.

To date, low-level phylogenetic relationships in non-model fungi have essentially been inferred using a small number of phylogenetic markers: nuclear and mitochondrial rDNA (including the internal transcribed spacer [ITS] region of the nuclear ribosomal repeat unit largely used for taxa discrimination and subgeneric phylogenetic inference [5], [6]), β-tubulin, γ-actin, Chitin synthase I and Elongation factor EF-1α [7], [8], [9], [10], [11]. The popularity of these genes may be explained by historical and/or practical criteria, e.g. publication of the first “universal primers”, provided for a large range of fungal species (Figure 1). However, the rationale behind the selection of these genes is not always clear and using such freely available primers has already resulted in amplification and analysis of gene regions which yielded inaccurate phylogenetic signal for the subset of the desired taxonomic range (see, for example [12], [13]). Some of these genes may also be duplicated in several specific taxa and thus, have the potential of being phylogenetically discrepant [13], [14], [15], [16], [17], [18].

The increase in complete fungal genome sequencing has given rise to a new wave of model organisms, empowered by well-established genomic resources. Usefulness of these genomic resources for SNP or microsatellite markers development has largely been proven [19], [20], [21], [22]. Similarly, these resources have also been successfully used to infer kingdom-wide fungal phylogenies [18], [23], [24], [25] and occasionally to study relationships between close species (e.g. 69 markers available across 12 Saccharomycotina species; [25]). *Ad-hoc* phylogenetic informative markers from protein-coding loci can be obtained with these resources. Recently, a bioinformatic method named FUNYBASE was developed to identify and assess the performance of single-copy protein-coding loci for phylogenetic analyses in fungi [4]. This method involved automated BLAST comparisons of whole genome sequences to identify single copy protein-coding homologs (SCPCH) among 21 fungal genomes and assessed their informative value for phylogenetic reconstruction i.e. their performance in recovering a reference species tree reconstructed from concatenated protein alignments [4], [7]. Several of these genes proved to be successful to reconstruct species trees in specific fungal genera, including *Botrytis* and *Penicillium* [7].

The utility of these genomic resources for making phylogenetic inferences in the vast majority of non-model fungal species remains a largely unexamined question. Relatively few studies have investigated (with success) selection approaches of sequences homologous to those found in the genome sequences of fungal model species to develop PCR primer-based markers in non-model-ones [9], [26], [27], [28]. Cross utilization of genomic tools to infer phylogenies in non-model organisms requires resolution of a fundamental conflict between the need to identify genomic sequences that are conserved (largely or wholly) across many divergent taxa, and the need to identify DNA-level differences that reflect the evolutionary history at the desired taxonomic level. Identifying particular loci with such desirable properties among thousands of potential loci available in model organisms may appear somewhat impractical or at least daunting.

We introduce a bioinformatic approach to automatically screen genomic databases (about 107 fungal genomes fully completed or in the draft assembly stage on February 2011 [see http://fungalgenomes.org/genome]) for SCPCH that might be well suited for phylogenetic studies, specifically at lower taxonomic level in non-model fungal species (e.g. closely related species or even within species). Ideally, good phylogenetic SCPCH will be chosen to maximize the three following criteria: i. to use single copy nuclear genes; ii. to obtain successful and reliable PCR amplifications (and subsequent DNA sequencing) for all target taxa; and iii. to maximize the number of DNA-level polymorphisms in the amplified fragment. To achieve this last point, we developed a graphical evaluation system based on the estimation of nucleotide divergence (measured by nucleotidic diversity, π) relatively to substitution type (transition/transversion ratio [Ti/Tv]) [29]. We postulated that gene regions highly sensitive to substitutions (i.e. highly polymorphic “mutation hot-spots” [30]) could be potentially informative to resolve relationships between closely related taxa. We expect that a low %Ti (relatively to Tv) combined with a high divergence level observed between distantly related sequences would be an accurate indicator of such “mutation hot-spots”, since Tv gradually outnumbers Ti as two sequences diverge from a common ancestor.

We implemented the bioinformatics approach in a single executable application named “PHYLORPH” (PHYLogenetic markers for ORPHan species), developed in Python language (ver. 2.6) and freely available. As test case, we applied it to the development of phylogenetic SCPCH sets in four groups of non-model fungal plant pathogens. We experimentally verified the efficiency and success of the method by i. amplifying and sequencing some of the SCPCH proposed by PHYLORPH and ii. ascertaining that the selected markers exhibited enough variation to resolve relationships within the groups of non-model fungi considered. The amplification success and levels of polymorphism in these SCPCH was compared with those obtained for a panel of genes traditionally used to build fungal phylogenies (e.g. nuclear rDNA, β-tubulin, γ-actin, Elongation factor EF-1α, Chitin-synthase). In addition, the phylogenetic performance of some of these SCPCH was ascertained by sequencing additional taxa and comparing phylogenies with ITS reference trees.

## Results

### Computational testing

The first step in the PHYLORPH method is the identification of single copy protein-coding homologs (SCPCH) by performing a blast search with a protein dataset retrieved from FUNYBASE, PHYLOME-T60 or OrthoMCL-DB against several fungal genomes (among the 107 available with full sequence; Table S1), close to the non-model taxa under investigation. In order to test the universality of our method, we performed 40 PHYLORPH runs, with one species dataset out of the 21 available in FUNYBASE blasted against two to five whole genomes in each run (Table S2).

**Figure 2:**
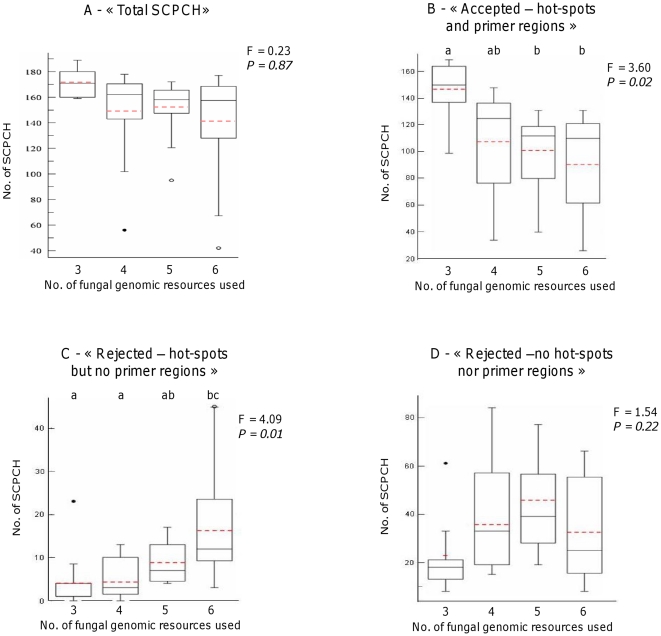
Number of candidate SCPCH obtained in 40 different PHYLORPH runs. The distributions are represented with boxplot depending on the number of fungal genomic resources used (3 to 6 i.e. one species dataset from FUNYBASE and 2–5 full genome sequence; see Table S2) in the different runs. Boxplot representations include the median (central bar), the mean (dotted red line), the position of the upper and lower quartiles (called Q1 and Q3; central box), extremes of the data (‘‘whiskers’’) and very extreme points that can be considered as outliers (dots; points that exceed Q1+1.5IQR and Q1-1.5IQR for the upper and lower parts of the distribution, respectively, where IQR = Q3-Q1). Letters above boxplots indicate significant differences between groups as determined by a Tukey’s HSD test after significant one-way ANOVA (F and P-values on the right). “Accepted” are candidates SCPCH which contain both «mutation hot-spot» and «priming site» regions; « Rejected – no primers » are those which contain only « mutation hot-spot » region(s); « Rejected » are those with no «mutation hot-spot» nor « priming site » regions; “Total” includes all the SCPCH (“Accepted” + « Rejected – no primers » + « Rejected ») retrieved in the searches.

The first objective of this test was to examine the variation in the number of SCPCH obtained relatively to the number of genomes considered. PHYLORPH proposed 152 (±30) single-copy orthologs on average over the 40 requests. This value did not significantly decrease when new genomic resources were gradually added, suggesting that as long as less than 6 genomic resources are considered, the number of SCPCH found between genomes strive for a constant value (Figure 2A). Interestingly, 119 SCPCH out of the 246 of FUNYBASE were systematically retained among 30 of the 40 searches. In contrast, 21 SCPCH out of the 246 were not retained in any of the 40 searches, indicating that these SCPCH were either absent or found as multiple copies in the genome sequences examined (Figure S1).

The second objective aimed to test the accuracy of the sliding window analyses. Whatever the request made and the number of genome sequences used, PHYLORPH proposed a large panel of “Accepted” SCPCH i.e. candidate SCPCH which contain at least a “mutation hot-spot” region flanked by two “priming site” regions i.e. nucleotide regions sufficiently conserved for primer design (Figure 2B). The average number of “Accepted” SCPCH proposed was 110 (±37) and the values ranged from 26 for a request made with the 246 FUNYBASE proteins of *Ustilago maydis* on the genomes of *Puccinia graminis*, *P. triticina*, *Melampsora larici-populina*, *Sporobolomyces roseus* and *Cryptococcus neoformans* to 169 for the 246 proteins of *Phanerochaete chrysosporium* requested on *Coprinopsis cinereus* and *Pleurotus ostreatus*. We observed a significant decrease in the mean number of “Accepted” SCPCH when the number of genomic resources considered in a PHYLORPH run was increased (Figure 2B).

In addition, the relatedness between the genomes used in the request seemed to influence the number of “Accepted” SCPCH. Mean genetic diversity (measured by π; [31]) observed between the genome sequences was negatively correlated with the number of “Accepted” SCPCH (r = -0.61, P<0.0001) and positively correlated with the number of SCPCH categorized into the “Rejected-no primers” folder (r = 0,74 P<0.0001; data not shown). The observed decrease in the number of “Accepted” SCPCH was thus likely linked in a large part to the growing difficulty to identify “priming regions” when an increased number of (distantly related) genomic resources was considered (Figure 2C). In counterpart, the identification of regions of maximum nucleotidic variation (“mutation hot-spots”), did not seem to be significantly impacted by the increase of the number of genomic resources (Figure 2D). These “mutation hot-spot” regions were well represented, since they represented 19.4% on average of the total length of all “Accepted” SCPCH (data not shown).

### Experimental validation

To assess the reliability of our method, we carried out an experimental validation by amplifying and sequencing a sample of proposed markers in four different groups of non-model fungi: *Erysiphe alphitoides* and *E. quercicola* (oak powdery mildews), North American and Eurasian lineages of *Heterobasidion annosum*, individuals of *Armillaria ostoyae* and cryptic species of the *Hymenoscyphus albidus*/*H. pseudoalbidus* (anamorph *Chalara fraxinea*) complex.

#### PCR and sequencing success

For each group of non-model species tested, the primer pairs for 10 to 14 of the SCPCH proposed by PHYLORPH were tested for their capacity to amplify and sequence the expected DNA fragment. Based on observed sequence conservation/variation between *Blumeria graminis, Sclerotinia sclerotiorum* and *Botrytis cinerea*, PHYLORPH proposed 133 “Accepted” SCPCH for oak powdery mildews. We performed experimental validation on a panel of 12 SCPCH. After “classical” PCR with annealing at 55°C, two candidate SCPCH yielded amplification products of the expected size in *E. alphitoides*/*E. quercicola* (FUNYBASE ID MS447 and MS294). We then attempted to sequence additional SCPCH that yielded multiple bands at the first PCR stage by using PCR touch-downs or gel extraction to isolate the appropriate size band. PCR touch-downs resulted in two new amplification products among which only one (MS550) yielded readable sequencing chromatographs without mixed peaks (Table 1). In all except one (MS393) of the three gel extraction attempts, we were mostly unsuccessful since sequencing yielded either poor quality sequences (FG1020) or good sequence but unexpected fragment homologies (FG534). In this last case, we suspected that cross-amplification with DNA from the host had occurred since the sequencing reaction yielded products of strong homology with plants. PCR amplification efficiencies obtained for the candidates SCPCH tested on oak powdery mildews were compared with those obtained for universal primer pairs for genes commonly used in fungal phylogenies (EF-1α, Calmodulin, Chitin synthase I, γ-actin, Histone-3 and -4 and β-tubulin). Globally, the universal primer pairs did not perform better than the PHYLORPH genes, with only three out of ten yielding a single PCR fragment (Table 2). Morever, direct sequencing of these PCR products resulted either in jammed sequencing chromatographs (primer pairs CHS-79F/CHS-354R and Bt1a/BtMycR_Ery) or in one single readable sequencing chromatograph which exhibited high sequence similarity with a plant β-tubulin gene (primer pair Bt1a/Bt1b, best blastn hit: 89% identity with a *Populus trichocarpa* tubulin beta chain, Ac. No. XM_002330576). To assess the possibility of multiple copies of the β-tubulin gene in oak powdery mildew genomes, we performed a direct cloning of the PCR products obtained with primers Bt1a/BtMycR_ery for one *E. alphitoides* and one *E. quercicola* strain (data not show). Selective amplification and sequencing of ten clones in each strain resulted in only one allele for the *E. quercicola* strain whereas two alleles (81% identity) were obtained for *E. alphitoides*, indicating the possible occurrence of at least two divergent copies for the β-tubulin gene in this last species or contamination with material from other fungi as oak powdery mildew hyperparasites [32].

**Table 1:** PCR and sequencing results for the SCPCH candidates tested across the four non-model systems used as test cases.

	Development						Optimization						
Non-model systems	PHYLORPH		PCR 40 cycles with annealing at 55°C				Touch-down 50–55°C				Band excision		Total No. SCPCH sequenced (%)
	No. SCPCH targeted	No. primer pairs developed	0 band	1 band	>1 band	No. SCPCH sequenced^1^	0 band	1 band	>1 band	No. SCPCH Sequenced^1^	No. tried	No. SCPCH Sequenced^1^	
*E. alphitoides*	12	16	3	2	7	2	3	2	5	1	3	1	4 (33.3)
*E. quercicola*	12	16	3	3	6	2	5	2	2	1	3	1	4 (33.3)
*H. albidus*	14	21	-	-	-	-	13	1	0	1	-	-	1 (7.1)
*H. pseudoalbidus*	14	21	-	-	-	-	13	1	0	1	-	-	1 (7.1)
*H. annosum* North American type	10^2^	10	4	1	5	1	5	3	1	3	-	-	4 (40.0)
*H. annosum* Eurasian type	10^2^	10	4	1	5	1	4	5	0	3	-	-	4 (40.0)
*A. ostoyae*	10^2^	10	8	0	2	0	7	3	0	1	-	-	1 (10.0)

1 Implies that the SCPCH has successfully been sequenced and corresponds to the expected sequence product.

2 The same SCPCH/primer pairs were tested for *H. annosum* and *A. ostoyae*.

**Table 2:** PCR and sequencing efficiencies on oak powdery mildews, *Erysiphe alphitoides* and *E. quercicola*, obtained with fungal primers developed in Carbone and Kohn (1999), Glass and Donaldson (1995) and Einax and Voigt (2003).

Reference	Primer pair	Gene	PCR 40 cycles – annealing 55°C		Sequencing product(s)
			1 band	> 1 band	
Carbone and Kohn, 1999	EF1-728F/EF1-986R	Elongation factor EF-1α	-	-	-
	CAL-228F/CAL-737R	Calmodulin	-	-	-
	CHS-79F/CHS-354R	Chitin synthase I	√	-	Multiple sequences
	ACT-512F/ACT783R	γ-actin	-	√	-
Glass and Donaldson, 1995	H3-1a/H3-1b	Histone-3	-	-	-
	H4-1a/H4-1b	Histone-4	-	√	-
	Bt1a/Bt1b	β-tubulin	√	-	One single sequence – Homolog to plant β-tubulin
Einax and Voigt, 2003	F- βtub3^1^/F- βtub2r^1^	β-tubulin	-	-	-
	F- βtub3^1^/F- βtub4r^1^	β-tubulin	-	-	-
This study	Bt1a/BtMycR_ery^1^,^2^	β-tubulin	√	-	Multiple sequences homolog to fungal β-tubulins

1 Degenerate oligonucleotide primer.

2 Reverse primer developed in the Bt1b primer region to only target fungi and avoid amplification of plant β-tubulin.

For *H. annosum* and *Armillaria ostoyae*, PHYLORPH supplied 31 “Accepted” SCPCH among which 10 were experimentally tested (Table 1). For *H. annosum*, four candidates (FG821, FG909, FG975 and FG533) yielded PCR and sequencing success. Sequencing of one candidate (FG543) was correctly achieved for *A. ostoyae* although three had resulted in PCR success, each with one single product amplified with the PCR touch-down protocol. The PCR fragment lengths and the sequences obtained for the two other candidates (FG649 and FG756) were not homologous to the expected SCPCHs.

Finally, for *H. albidus*/*H. pseudoalbidus*, the search made with the 246 protein sequences of *S. sclerotiorum* on the genomic sequences of five fungi included within Leotiomycetes or Sordariomycetes classes, resulted in 76 “Accepted” SCPCH. Only one candidate was validated out of the 14 tested with 21 primer pairs (Table 1).

#### Polymorphism levels

**Figure 3:**
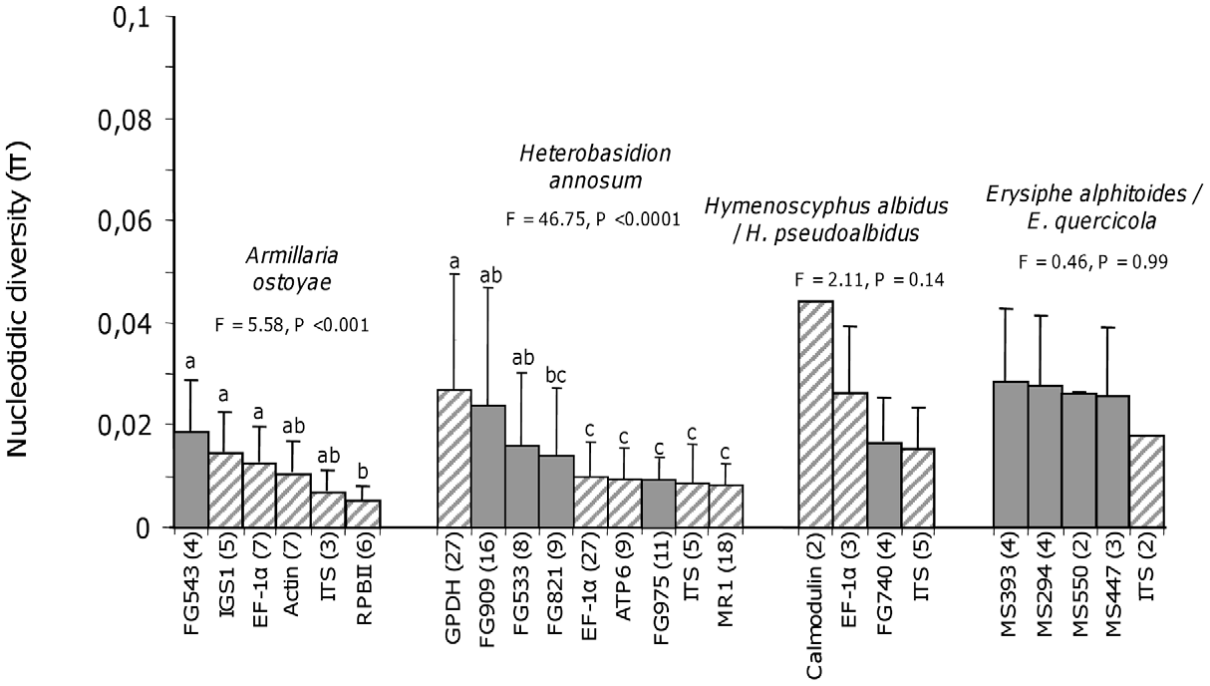
Nucleotidic diversity levels obtained for the PHYLORPH genes. The nucleotidic diversity levels obtained for the PHYLORPH genes (identified on the abscissa with their FUNYBASE ID) in the four non-model system considered are compared with those obtained for loci commonly used in multigene phylogenies for the same systems (retrieved from the NCBI database: Actin, γ-actin gene; ATP6, mitochondrial ATP synthase subunit 6 gene; Cal, Calmodulin gene; EF-1α, Elongation Factor-α1 gene; GPDH, Glyceraldehyde-3-phosphate dehydrogenase; IGS, Intergenic spacers I of the nuclear rDNA; ITS, internal transcribed spacer of the nuclear rDNA; MR1, MRI, portion of two non-homologous mitochondrial rDNA insertions in the ML5–ML6 region; RPBII, largets subunit of the RNA polymerase II gene). In plain grey, PHYLORPH genes; In dashed grey, loci commonly used in multigene phylogenies. Letters above columns indicate significant differences between nucleotidic diversity means as determined by a Tukey’s HSD test after significant one-way ANOVA.

The DNA sequences obtained were then evaluated for polymorphism and their ability to resolve taxa relationships in the four non-model species groups was studied. For each of the four non-model systems considered, the SCPCH sequences contained enough variation to resolve taxa relationships. In the *E. alphitoides*/*E. quercicola, H. albidus*/*H. pseudoalbidus* and *H. annosum* groups, inter-specific (or inter-types for *H. annosum*) polymorphisms ranged from 0.8 to 5.2%, depending on the SCPCH considered (Table 3). In *A. ostoyae* an intra-specific polymorphism level of 2.6% (eight SNPs) was found for the gene FG543 (Table 3). To compare these levels with those obtained for loci commonly used in multigene phylogenies, we retrieved 14 sequences datasets from the NCBI database (http://www.ncbi.nlm.nih.gov) representing eight different loci (IGS1, Elongation factor EF-1α, γ-actin, ITS, RPBII, GPDH, ATP6 and MR1; Figure 3). These datasets were manually curated to retain only sequences that corresponded to the divergence levels considered in the four experimental validations (i.e. *E. alphitoides*/*E. quercicola* in different populations in France, *H. albidus*/*H. pseudoalbidus* for different populations from France, NA vs. EU types in *H. annosum* and at a regional scale from different populations in *A. ostoyae*). In each non-model system, the PHYLORPH genes were generally ranked among the best-performing ones in term of nucleotidic diversity. Systematically, these SCPCH showed higher nucleotidic diversity than the loci expected to show extensive nucleotide variation as the ITS regions [33] or mitochondrial genes (rDNA and ATP6) for *H. annosum* [34], [35] (Figure 3). In all except one case (FG543 in *A. ostoyae*), polymorphism levels were on average one and a half higher in predicted “mutation hot-spot” regions than in the full SCPCH length (Table 3). An average polymorphism value of 5.7% was found in “hot-spot” regions, whereas this value was 3.75% on average for the full SCPCH sequence. Remarkably, 29.4% (ten out of 34) of the SNPs found in MS294 for oak powdery mildews were located in “mutation hot-spots” even though these regions represented only 15.2% (100 nt) of the SCPCH sequenced part.

**Table 3:** Polymorphism results for the SCPCH candidates tested across the fungi panel.

		Sequence attributes				Polymorphisms^1^					
	FUNYBASE ID – homology	Length	No. strains sequenced	No. “hot spots” regions (length)	No. of introns (length)	No. SNP (%)	Indels	No. SNP in “hot spots” (%^2^)	No. SNPs in intron(s)(%^3^)	Ti/Tv	dN/dS
*E. alphitoides*/*E.quercicola*	MS294 - Nicotinate phosphoribosyltransferase	656 nt	10/10	3 (100 nt)	1 (48 nt)	34 (5.2%)	-	10 (10%)	3 (6.2%)	27/7	10/21
	MS447 - Dihydrouridine synthase	409 nt	9/9	3 (120 nt)	-	14 (3.4%)	-	5 (4.2%)	na	7/7	6/8
	MS550 - Phosphatidylglycerolphosphate synthase	216 nt	5/8	3 (110 nt)	-	10 (4.6%)	-	3 (4.5%)	na	8/2	4/6
	MS393 – tRNA methyltransferase	448 nt	9/9	4 (220 nt)	-	22 (4.9%)	-	11 (5.0%)	na	15/7	13/9
*H. albidus*/*H.pseudoalbidus*	FG740 - Cobalamin-independent methionine synthase	569 nt	15/15	4 (105 nt)	-	13 (2.3%)	-	5 (4.8%)	na	10/3	2/11
*H. annosum* NA type/EU type	FG821 - GPI-anchor transamidase precursor	418 nt	1/15	2 (55 nt)	2 (145 nt)	9 (2.2%)	-	2 (3.6%)	6 (4.1%)	4/5	0/3
	FG909 - Centromere/microtubule binding protein cbf5	656 nt	1/15	0	2 (115 nt)	15 (2.3%)	-	na	4 (3.5%)	12/3	1/10
	FG975 - Fimbrin actin-bundling protein	496 nt	1/11	0	1 (69 nt)	4 (0.8%)	1 (4 nt)	na	1+1 gap (2.9%)	5/0	0/5
	FG533 - histone acetyltransferase	901 nt	1/11	0	4 (218 nt)	20 (2.2%)	2 (3 nt)	na	9 (4.1%)	16/4	0/20
*A. ostoyae*	FG543 - F1-ATPase subunit gamma	312 nt	4	2 (85 nt)	2 (110 nt)	8 (2.6%)	-	2 (2.4%)	5 (4.5%)	6/2	0/3

1 Inter-specific polymorphisms are considered for the *E. alphitoides*/*E. quercicola*, *H. albidus*/*H. pseudoalbidus* and *H. annosum* NA/EU groups; intra-specific polymorphisms are considered for *A. ostoyae*.

2 % relative to the total length of “hot spot” regions.

3 % relative to the total length of intron regions.

A total of 11 putative introns were predicted in the five SCPCH successfully amplified for *A. ostoyae* and *H. annosum.* After sequencing, ten of the predicted introns were retrieved; one out of the three introns predicted in FG533 was not retrieved in sequences obtained for *H. annosum* at this locus. As expected for protein-coding sequences, intron regions generally contained more polymorphisms than exons; indel polymorphisms (one to four nucleotides long) were exclusively found in these regions (Table 3).

#### Phylogenetic performance of SCPCH

In this third test, we verified the phylogenetic performance of the SCPCH obtained for oak powdery mildews and *H. albidus*/*H. pseudoalbidus* by sequencing these genes in additional related species, reconstructing phylogenies and assessing their congruence and resolution with respect to ITS reference trees.

**Figure 4:**
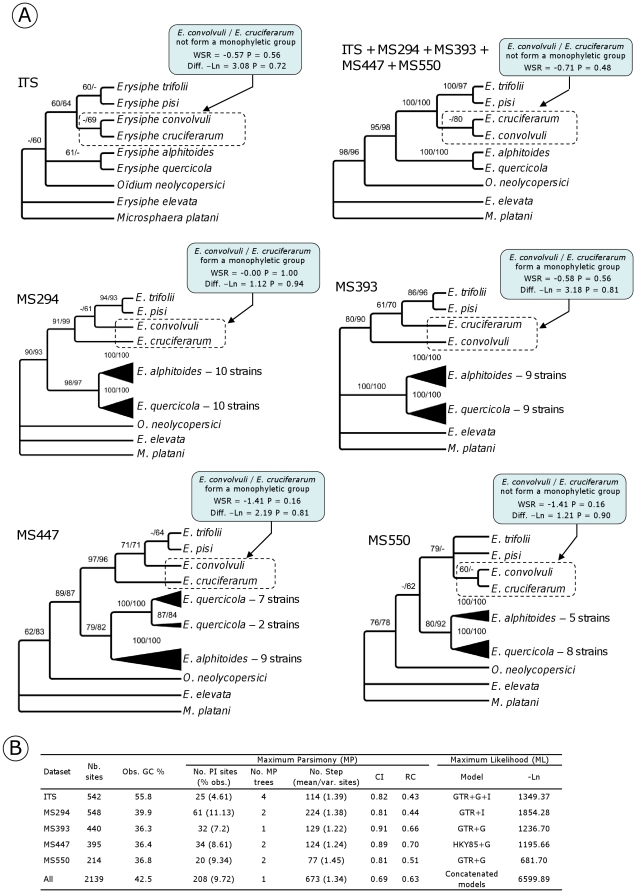
Phylogenetic comparisons between the ITS and the PHYLORPH genes obtained for oak powdery mildews and five related species. (A) Bootstrap values ≥60% are given above each node as follows: MP bootstrap value/ML bootstrap value. Boxes with arrows indicate the constraint nodes applied to the different datasets and results of both WSR- and SH-tests following constrained and unconstrained tree comparisons. Table (B) contains alignment lengths, summary tree statistics issued from MP analyses, and ML models; PI, Parsimony informative sites; CI, consistency index; RC, Rescaled consistency index.

The four SCPCH obtained for oak powdery mildews were amplified/sequenced in seven additional powdery mildew species (Figure 4). The four genes were successfully amplified for the different species, except MS393 in *Oïdium neolycopersici.* Although these genes and ITS evolved at similar rates (1.22 to 1.38 steps/site vs. 1.39 steps/site respectively), and exhibited equal levels of homoplasy (CI = 0.81 to 0.91 for the PHYLORPH genes vs. CI = 0.82 for ITS), the phylogenetic content of SCPCH appeared slightly higher than ITS. MP (maximum of parsimony) heuristic searches resulted in one or two trees, whereas four equal trees were found for ITS. The majority rule consensus tree obtained in MP and ML (maximum of likelihood) for ITS appeared unresolved and weakly supported with values of node confidence which never exceeded 70%. In contrast, an enhanced phylogenetic resolution was observed for the trees obtained with the PHYLORPH SCPCH which showed three (MS550) to seven (MS447) nodes supported with bootstrap values ≥70%. Interestingly, each gene resolved a monophyletic group for the oak powdery mildew species complex (bootstrap support varying from 79 to 100%), which was weakly supported in the ITS tree (61% in the MP tree). Only one topology incongruence implicating the *Erysiphe convolvuli* and *E. cruciferarum* taxa was found between ITS/MS550 and MS294/MS393/MS447 trees. WSR-tests in MP and SH-tests in ML of alternative constraint hypotheses (i.e. *E. convolvuli*/*E. cruciferarum* clustered in an exclusive group for MS294, MS393 and MS447 and *E. convolvuli*/*E. cruciferarum* not clustered for ITS and MS550) indicated that this conflict was not significant. The combined ML analysis of the ITS, MS294, MS393, MS447 and MS550 supported the resolution of one monophyletic clade for *E. convolvuli*/*E. cruciferarum* (Bootstrap support = 80%).

**Figure 5:**
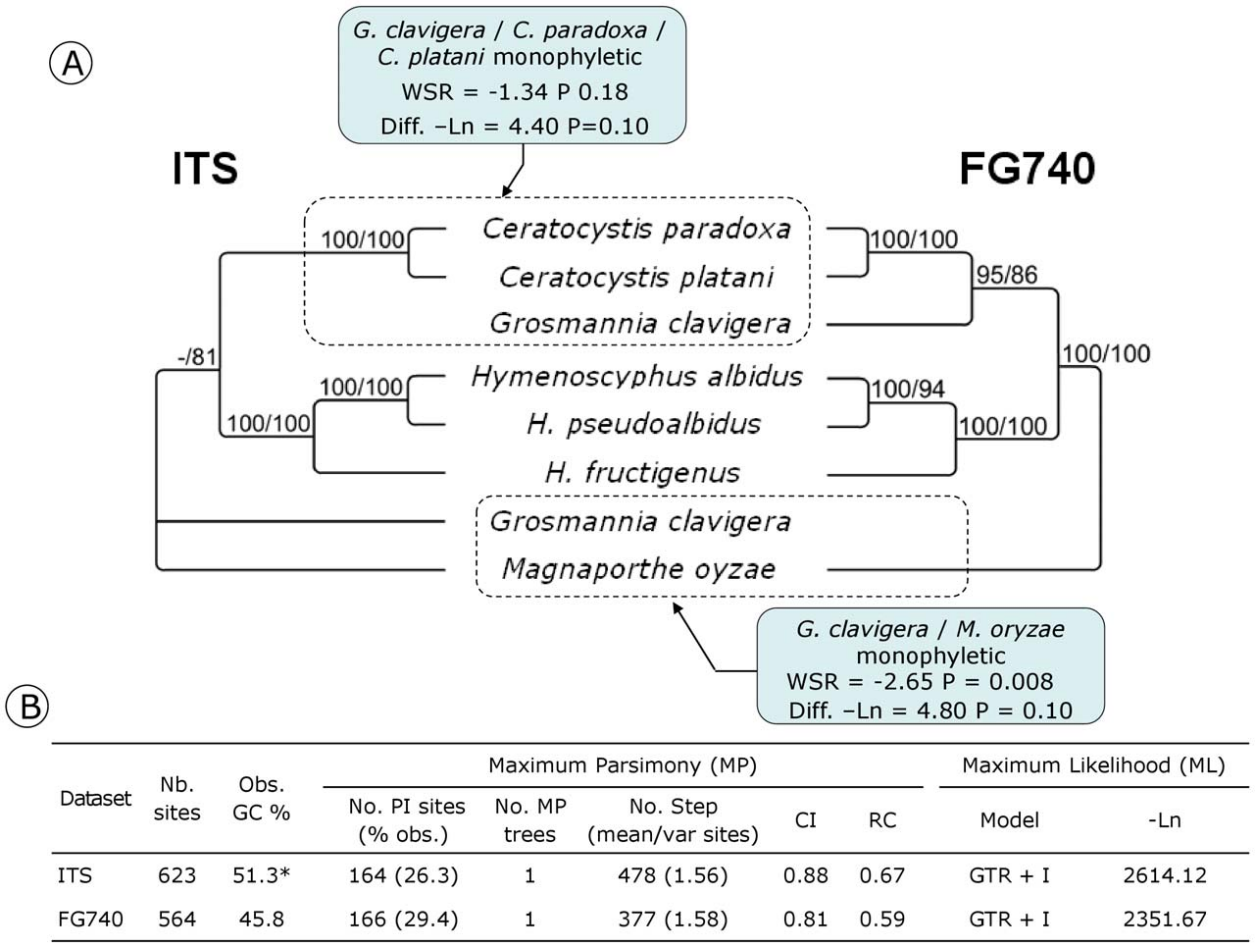
Phylogenetic comparison between the ITS and the PHYLORPH gene (FG740) obtained for *H. albidus*/*H. pseudoalbidus* and 6 related species. (A) Bootstrap values ≥60% are given above each node as follows: MP bootstrap value/ML bootstrap value. Boxes with arrows indicate the constraint nodes applied to the different datasets and results of both WSR- and SH-tests following constrained and unconstrained tree comparisons. Table (B) contains alignment lengths, summary tree statistics issued from MP analyses, and ML models; PI, Parsimony informative sites; CI, consistency index; RC, Rescaled consistency index. The asterisk indicates a significant difference in base frequencies across taxa (Chi-2 = 70.29, df = 18, P<0.0001).

The FG740 SCPCH was sequenced in four species related to *H. albidus*/*H. pseudoalbidus* (Figure 5). Both the FG740 MP and ML trees were fully resolved (five nodes vs. four for ITS) with strong node confidence values up to 85% (Figure 5). A topological conflict implicating *Grosmannia clavigera* was detected between the ITS and FG740 trees. Monophyly of *G. clavigera*/*C. platani*/*C. paradoxa* was not significantly rejected by the ITS dataset. In contrast, the basal position of *G. clavigera* was rejected in MP by the FG740 dataset (P = 0.008). Interestingly, the ITS dataset exhibited significant heterogeneity in base composition among taxa (Chi-2 = 70.29, P<0.0001). The high GC frequencies observed in ITS for G. *clavigera* (GC content = 64%) and *M. oryzae* (54%) relatively to other taxa (43 to 51%) have likely tended to group these unrelated species, explaining the unexpected basal placement of *G. clavigera* [36]. Finaly, the topology of the FG740 gene was consistent with classification of *G. clavigera*, *C. platani* and *C. paradoxa* within the Ophiostomatales order [37].

## Discussion

Our results indicate that the isolation of numerous SCPCH in several fungal species with or without a fully sequenced genome should be rapid and efficient in nearly any Ascomycota and Basidiomycota fungus. The large number of new candidates extracted using this method should help to resolve the inherent problems associated with phylogenetic inferences made from single gene trees and limitations of the most commonly loci sequenced to reconstruct multigene phylogenies [1]. Few studies have previously examined similar approaches, by comparing genomic resources from related species to develop phylogenetically informative markers in non-model species (reviewed in [38]). For the fungal kingdom, Aguileta et al. [4] initially evaluated the phylogenetic performance of single copy protein-coding homologs (SCPCH) retrieved among 21 genomes with the goal of providing a reliable resource of orthologous gene families (246 SCPCH regrouped in the FUNYBASE database [7]) to perform phylogenetic analyses. With PHYLORPH, we aimed to help researchers not specifically interested in genomics and bioinformatics to gain genetic information on their studied species or group of species. Indeed, PHYLORPH increases the interest which previously aroused the identification of the 246 FUNYBASE SCPCH by routinely expanding their use to a total of 107 fully sequenced fungal species and by demonstrating that some of these genes are relevant to resolve relationships between closely taxa. Using the 246 FUNYBASE SCPCH avoids to systematically perform the cumbersome work needed to re-extract single-copy orthologous genes among the large amount of gene models available in each fungal genome and allows to directly target well-annotated and phylogenetically informative genes [4], [7].

The establishment of correct orthology and paralogy is crucial to infer accurate evolutionary relationships. If paralogs are inadvertently amplified, the resulting gene tree may not reflect the true species histories [3], [4]. Several orthology-prediction methods (and ensuing ortholog-databases) have emerged in recent years, among which the “pairwise comparison” and “phylogenetic” approaches remain the most popular [39], [40], [41]. These methods can be influenced by biases related to the lack of accuracy of gene predictions along genomes and the presence of multidomain proteins which can possibly generate artificial clusters of orthologs. To allow users to have access to an extended range of orthology repositories (and thus to consider a larger choice of potential markers), PHYLORPH can use the fungal proteins from the OrthoMCL-DB and PHYLOME-T60 ortholog-databases [25], [42] as templates instead of those provided in FUNYBASE. These three ortholog databases differ in the methods followed to obtain the clusters of putatively orthologous genes. For instance, to identify the 246 single-copy orthologs of the FUNYBASE database, pairs of genes were automatically identified from complete fungal genomes using a “pairwise comparison” method (i.e. with the Bi-directional Best Hit method; BBH) and clusterized with the Tribe-MCL algorithm [43]. The BBH strategy was also used for OrthoMCL-DB but automatic detection of ortholog clusters was performed with the OrthoMCL algorithm [44], which is well-known to perform better than Tribe-MCL [45]. One-to-one orthologs regrouped into the PHYLOME-T60 database were inferred using a large scale phylogenetic approach. Such phylogeny-based method is considered more accurate than standard BBH strategies, since less prone to false positive ortholog linkages [39], [40]. Cross comparisons of these three different ortholog resources showed significant differences in the protein sets, indicating low redundancy between the databases. We expected that the reliability of databases on orthology is likely differ mainly because of the differences between the orthology predictions methods but also to differences in fungal species content and possibly asynchronous updates of the data resources [39]. Finally, these three ortholog databases represent a potential of 329 different markers (out of a 376) available among 62 fungal species (35 genera, 18 orders) (Figure S2).

The method of selection of single-copy genes implemented in PHYLORPH is also based on a BBH strategy, and thus could suffer of potential pitfalls of “pairwise comparison” methods. False positives are generated with BBH if the true ortholog was lost in the queried species, and the best blast hit is a paralog (i.e. mistaken for ortholog). In such a case, primers issued from PHYLORPH could either target the mistaken paralog resulting in gene/species tree discrepancies or multiple paralogous copies that will complicate amplification, sequencing and alignment. For these reasons however, primer pairs amplifying paralogs can easily be identified and discarded. Furthermore, integrating more accurate methods of orthologs prediction as those based on phylogeny will require reconstructing trees for each PHYLORPH request, and could drastically increase computation needs.

Globally, when integrating the three ortholog databases (FUNYBASE, OrthoMCL-DB and PHYLOME-T60) and the 107 full genome sequences available to date, the taxonomic coverage provided by PHYLORPH represents a potential of marker development for about 2247 known genera within the fungal kingdom (2079 within the dikarya subkingdom) [46]. In few rare cases however, the searches conducted with our program were limited by the inability to obtain genomic alignments for a set of two or more species representatives of the studied target group. For example, the Lecanoromycetes (estimated to be >13,500 species; about 90% of all described lichen-forming Ascomycota, [46]) cannot yet be used in PHYLORPH, since no model species is fully sequenced in this class. However, we expect that fungal taxonomic coverage will shortly be increased as long as new fungal genomes are sequenced, specifically with the thrust of new sequencing technologies applied to microbial full genome sequencing [47], [48].

Selecting efficient primers from sequenced model organisms to transfer in orphan species appears as one of the complicated step of the approach presented here. The substantial diversity in fully sequenced fungal genomes (and their availability in PHYLORPH; discussed above) provides the possibility to easily consider several genomic resources for a single search, promoting the identification of a maximum of mutations at each site (particularly, in conserved regions allowable for primer design) and thus increasing the confidence in primer design. Our computational tests however indicated, that increasing the number of genomic resources in a search can hastily reduce the number of candidate SCPCH proposed by PHYLORPH, due to the impracticability to automatically identify “primer regions” (with the default criteria implemented in the program). This problem can be increased if distantly related genomic resources are selected for the search, given that sufficiently conserved regions for primer design tend to rapidly decline. From a practical point of view, two general approaches are conceivable for designing oligonucleotide primers that are potentially useful across a broad selection of species [49]: a non-degenerate primers strategy that take into account nucleotide mismatches that form weak or partial bonds [50] or alternatively, the design of degenerate primers [49]. We considered this second approach in our experimental validation by designing partially degenerate primers in few candidates SCPCH (<15) obtained from model species which diverged from the test non-model species about 200–300 Million of years ago (Mya). According to the analyses of [38], PCR success obtained for marker transfers are correlated with the divergence times observed between the targeted non-model species and the reference model species. The PCR success rates obtained in our experimental validation varied from 7 to 40%, accurately corroborating the PCR efficiency values expected for divergence time greater than 200 Million years [38]. One of the main drawback of degenerate primer pairs however, is that only a fraction of the primer pool will contain perfect matches to the target sequences, resulting in low PCR amplification yields and sometimes cross amplifications of non-target DNA. In few cases of our experimental validation, the degenerated nature of the primers used, coupled with poorly-stringent PCR conditions likely resulted in such non-specific cross-amplifications. The occurrence of non-specific cross-amplifications may furthermore be enhanced in the specific case of biotrophic fungi (as powdery mildews), since fungal material obtained from these unculturable organisms can occasionally be contaminated with plant material (see for example, the two cases of our experimental validation [FG534 and β-tubulin] where DNA from the host plant was likely amplified and sequenced). Further optimisation of primer and PCR protocols could help to increase the proportion of markers that amplify the correct product across the species of interest (i.e. efficiency of the method) by avoiding these non-target amplifications [49]. However, defining and proposing alternatives for primer design and conditions to maximize PCR amplification specificity and success was beyond the scope of the experimental validation proposed in this work. Based on the fact that each PHYLORPH run usually provides many suitable SCPCH, we rather suggest adopting a marker development strategy based on testing several primer pairs with a standard PCR protocol on a large panel of candidate SCPCH. We expect that the few polymorphisms encountered in the alignment regions dedicated to primer design by PHYLORPH will be sufficiently representative of the group of species considered in the analysis. With many candidate SCPCH available for marker design, PCR and sequencing efficiencies are no longer a limiting factor since researchers can easily remove unsuitable candidates and move-on [38]. Furthermore, we rather recommend restricting searches made with PHYLORPH to a maximum of six genomic resources: one reference set extracted from FUNYBASE used to search the complete sequence of five related species (from the same class, as far as possible). These searches can be restricted in some cases depending on the number of whole genomes available in vicinity of the non-model species of interest. A minimum knowledge on the taxonomical position of the non-model species targeted is thus needed to allow an efficient selection of the genomic resources used in a marker search conduced with PHYLORPH. Interestingly, with these parameters, the primers obtained performed globally well when sequencing of the candidate SCPCH was extended to a larger number of taxa (test cases on species related to powdery mildews and *Hymenoscyphus* spp.). Thus, as expected, a substantial number of closely related taxa can be targeted using the primer regions provided by PHYLORPH, without requiring additional technical improvement. If required, a redefinition of primers based on alignment of the DNA sequences obtained and those used for the PHYLORPH search could help to extend this sequencing to more distantly related species.

A second challenge for PHYLORPH was to provide phylogenetic markers (candidate SCPCH) able to resolve phylogenetic relationships at low divergence levels. To evaluate the phylogenetic potential of each SCPCH proposed, we developed a graphical system which examines patterns of nucleotide substitutions using a sliding window analysis. We assumed that combined comparison of nucleotide divergence and substitution type observed between divergent species should help to localize “mutation hot-spots” (as measured by nucleotidic diversity [π] upper to %Ti). As we expected, the evolution rate obtained in these regions appeared to be sufficiently consistent to accurately discriminate closely related taxa. The identification of such “mutation hot-spot” regions was thus an attractive approach to get the maximum information for resolution in the case of recent divergences as those observed in species complexes. It remains however important to bear in mind that these rapidly evolving nucleotide sites could quickly be submitted to multiple hits, resulting in the loss of informative characters (i.e. homoplasy or saturation). The method we proposed aims to obtain phylogenetic markers for the resolution of recent divergences, between cryptic species in complexes or within genera. At these phylogenetic levels, limited saturation effects are expected [51]. Furthermore, gene phylogenetic informativeness can be considered as a tradeoff between optimal rate of evolution and effects of saturation [51], [52]. This relationship has been extensively discussed on theoretical grounds [53], [54], [55] and through empirical examples [29], [52], [56]. The work presented here does not intend to feed this debate, but rather to focus on practical and simple issues involved in obtaining the raw data for the desired phylogenetic analysis. Several methods including for example, character partitioning and exclusion have been proposed as means to enhance phylogenetic signal and eventually, accommodate with nucleotidic saturation [57], [58], [59].

Intron regions constitute an additional source of variation that can be considered when using PHYLORPH, since protein-coding sequences are targeted. Levels of variation found in introns appear adequate for use in both phylogenetic (e.g. [60]) and population genetic (e.g. [61]; [62]) studies. One of the main issues concerns the conservation of these regions (and subsequent transfer) over different taxa. Indeed, fungal species can vary in their intron distributions, from on average far less than one intron per gene for the yeasts *Schizosaccharomyces pombe* and *Saccharomyces cerevisiae* to an intron content among the highest known in eukaryotes in Zygomycota and Basidiomycota (4 to 6 per gene) [63], [64]. Specifically, although gene structure is known to be a slowly evolving phylogenetic character in fungi, intron composition can vary between closely related species as reported among *Cryptococcus* spp. [65]. Despite the experimental evidence that intron positions have remained conserved among the group of fungi tested in this study, correct predictions on their conservation among fungal lineage can remain hard to establish.

Whatever the sources of variation considered (mutation “hot-spots” or intron regions), enough polymorphisms were found to resolve phylogenetic relationships in the four non-model species groups studied. Three of these groups (*E. alphitoides*/*E. quercicola*, *H. albidus*/*H. pseudoalbidus* and *H. annosum*) were complexes of sibling and closely related species initially differentiated on the basis of polymorphisms found in their ITS sequences [66], [67]. The nine SCPCH obtained with PHYLORPH brought an additional deal of clarity in the taxonomical resolution of these complexes by providing polymorphism levels equivalent or greater than those found in the ITS region. For example, phylogenetic content of the FG740 gene was identical to those from three genes (including ITS) to recognize the existence of two distinct species, *H. albidus* and *H. pseudoalbidus*, the last one being the teleomorphic state of *Chalara fraxinea* [67]. Sequencing of this gene was extended to four additional species representing three genera (*Hymenoscyphus*, *Ceratocystis* and *Grosmannia*) and two orders (Helotiales and Ophiostomatales). The resulting dataset did not suffer from the base compositional bias observed in the ITS dataset and resulted in a well resolved tree, indicating the accuracy of this gene for phylogenetic reconstruction. In a similar way, the four SCPCH obtained for the oak powdery mildews will help to resolve phylogenetic relationships among cryptic species in this complex. Applying this criterion should be particularly useful in delimiting the sympatric lineages included within this complex [2]. When integrated in a larger phylogeny which included seven related powdery mildew species, these four SCPCH clearly delimited the oak powdery mildews species complex and accurately improved the initial phylogeny obtained with ITS sequences. In a fourth validation case, we attempted to obtain resolution at its most elementary-level by targeting intra-specific polymorphisms within the *A. ostoyae* species. One SCPCH was successfully amplified into the four isolates tested and contained intra-specific polymorphisms located either in predicted “mutation hot-spots” or intron regions. The polymorphism level obtained with this gene was upper than those found into the IGS regions known to be highly variable among (and even within) individuals of the same species (reviewed by [68]). PCR amplification and sequencing of additional isolates will help to determine if the sequence variation obtained with this marker is reliable for making population genetic studies.

Herein, we developed and evaluated an automated approach to mine fungal genomic resources and guide the selection of useful phylogenetic markers for clades of fungi in which only a few model organism genomes have been sequenced. Although we targeted a particular group of organisms (Ascomycota and Basidiomycota), our methods should be applicable to almost any group of organisms for which one or more complete nuclear genomes are available. This method thus successfully improves the toolbox of scientists interested in molecular phylogeny, phylogeography and taxonomy. Though faster and less expensive whole genome sequencing will be increasingly available in the near future [38], [48], [69], [70], cross-species primers as those obtained with PHYLORPH should still be useful for species for which full genome sequencing is not of interest or for which the available molecular techniques are found to be convenient to obtain the sample (biotrophic and endophytic fungi, for example). Furthermore, our marker development method only relies on standard molecular biology tools and on a simple and customized bioinformatic pipeline and is thus expected to be easily and routinely useable in any laboratory interested in a fast and cheap marker development procedure.

## Materials and Methods

### Overview of the method

**Figure 6:**
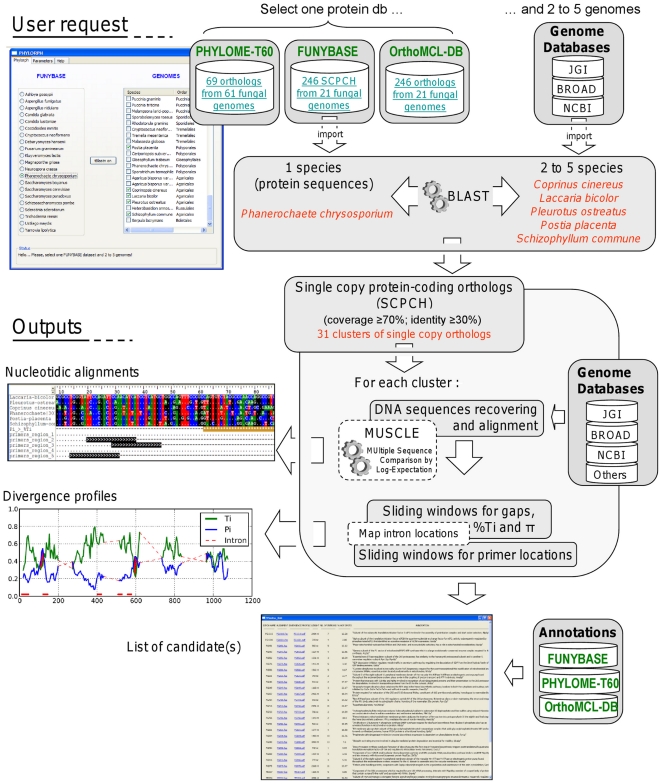
Overview of the bioinformatic procedure implemented in PHYLORPH. An explanatory example is presented with the request made for *Armillaria ostoyae* and *Heterobasidion annosum*.

The procedure can be divided into three steps: Step 1 is the identification of single copy protein-coding homologs (SCPCH) by performing a tblastn search with a protein dataset retrieved from FUNYBASE against different fungal genomes, close to the non-model taxa under investigation. FUNYBASE provides a subset of 21 genomes representing the major fungal taxonomic groups across a large phylogenetic scale, among which 246 clusters of single copy proteins were identified (Table S1). For each of these clusters, the FUNYBASE also contains information regarding the amino-acid model that best fit the data, an annotation (when available), the mean identity percentage of the species-sequences in the cluster, the number of the variable sites and a topological score i.e. a measure for performance of each gene in estimating the phylogeny of the species included in the cluster [4], [7]. To explore other fungal orthology repositories than FUNYBASE and offer a larger choice of potential phylogenetic markers, two additional protein datasets (PHYLOME-T60 and OrthoMCL-DB) are available for performing the identification of SCPCH throught tblastn searches. PHYLOME-T60 and OrthoMCL-DB contains 69 and 61 one-to-one protein orthologs across a range of 61 and 18 fungal species, respectively (Table S1). Step 2 consists in recovering and aligning nucleotidic sequences of the SCPCH selected in step 1. In step 3, a sliding window analysis is carried out on each alignment to identify the best candidates i.e. with i. appropriate evolutionary rate for being potentially useful in phylogenetic analyses among close taxa and ii. conserved sequence regions to allow development of degenerate primers. Candidate SCPCHs are categorized according to their content in priming sites flanking phylogenetic informative regions. These three steps are automated in a Python (ver. 2.6) application named PHYLORPH and controlled with a graphical user interface (GUI) developed using the WxPython module. Figure 6 gives a schematic overview and an explanatory example of the entire nucleotide protein coding loci discovery procedure. The executable version (2.0) of PHYLORPH can be downloaded at the following address: https://www4.bordeaux-aquitaine.inra.fr/biogeco_eng/Resources/Softwares/PHYLORPH.

#### Step 1: identification of single-copy orthologs

In order to increase the probability to obtain suitable DNA amplification of the targeted marker, the philosophy underlying PHYLORPH is to target the most closely related genomic resources available (same class or order, as possible) to the non-model species targeted. The PHYLORPH GUI allows the user to select one species protein dataset out of those available in FUNYBASE, OrthoMCL-DB or PHYLOME-T60 to BLAST (tblastn) against 2–5 genome datasets among a panel of 107 fully sequenced genomes (representing 28 orders among 14 classes within the fungal tree of life; Table S1). Upon user request (selection of one protein dataset and at least two full genome sequences located in the same class or order than the non-model species targeted), genomes are automatically downloaded from various sources (NCBI, JGI, BROAD and others; Table S1), formatted for tblastn searches and interrogated for similarities with the protein sequences of the species dataset selected. Tblastn results were considered non-spurious if they met three criteria (identical to those employed in FUNYBASE): i. coverage of at least 70% of the query protein, ii. identity of at least 30% and iii. e-value cutoff of 1e-20. Furthermore, these tblastn results are filtered-out for hits with potential paralogous copies. This step is automatically accomplished by simply discarding any SCPCH that significantly matched more than one genome location.

#### Step 2: alignment of nucleotide sequences

Phase 2 aims to provide a nucleotidic alignment for each SCPCH retained up to this point. First, the nucleotide-level records associated with the protein sequences of each SCPCH are automatically extracted from the previously selected genomes. Second, the files obtained are automatically aligned using the MUSCLE (version 3.7) algorithm [71]. Since the tblastn searches (i.e. search translated nucleotide database using a protein query) performed in phase 1 to identify SCPCH (Step 1) were against translated genomic sequences, any continuous run of protein sequence that scored a significant match had to represent an individual exon or portion thereof. This information coupled together with MUSCLE gap-opening parameter set at −1000, allowed accurate alignments of intron-exon boundaries.

#### Step 3: sliding windows

Patterns of nucleotide substitutions are then examined across each SCPCH alignment using a sliding window analysis. A 20 bp window with a 5 bp-step was chosen (default parameters), which improved visualization of nucleotide changes along the alignment independently of alignment length. Mean nucleotidic diversity (π; [31]) and percent transitions (%Ti) are graphed together to identify potential “mutation hot-spots”. These “mutation hot-spots” i.e. optimal regions for phylogenetic analysis among closely related taxa levels are identified as alignment regions which have a nucleotidic diversity greater than the %Ti [29]. We used %Ti rather than the more standard Ti/Tv ratio, to eliminate the problem of undefined values when transversions equal zero. In an attempt to compensate for possible differences in base compositions among genes (and allow %Ti to be comparable), each mutation type is divided by the count of its original base (e.g. a substitution from ‘A’ to ‘T’ divided by the number of ‘A’). This normalization of the Ti/Tv ratio is proposed as a default option of PHYLORPH. When at least one “mutation hot-spot” is detected, conserved alignment blocks suitable for primer development are identified using a method inspired from the strategy implemented in CODEHOP [72]: the DNA alignment is scanned in the 3’–5’ direction to identify a region of five nucleotides which starts on an invariant 3’ position and which has at most one SNP, followed by a region of five nucleotides with at most two SNPs and finally, a region of eight nucleotides with at most three SNPs. Distance parameter between forward and reverse priming sites can be adjusted by the user (default: 400 nt). Finally, SCPCH alignments (and graphs) that do not contain at least one “mutation hot-spot” region are automatically moved in a folder named “Rejected”. A second folder named “Rejected – no primers” holds those which contain at least one hot spot region but no conserved alignment blocks suitable for primer development. All the other alignments which contain at least one “hot spot” region flanked by two conserved alignment blocks are moved in a third folder named “Accepted”. Candidate SCPCH enclosed into the “Accepted” folder are listed onto an html window and saved in a tabulation-delimited file. These results are provided in a tabular view: the columns headers of the table are ID of the gene candidates, links towards fasta format alignment and divergence profile, alignment length, GC content, introns number and functional annotation of the candidate.

### Computational testing

We performed a total of 40 different PHYLORPH runs, including those made for the experimental validation as a test case. For each run, the approach previously described was strictly respected: one species dataset out of the 21 available in FUNYBASE was randomly selected and used to interrogate two to five (depending on the run; see Table S2) closely related genomic resources available in PHYLORPH (same class or order, as possible). We limited these tests to a maximum of six genomic resources (i.e. one dataset from FUNYBASE and five full genome sequences) to save computational time and avoid making searches between increasingly distantly related species which could increase potential for sequence mis-alignements. For each run, the number of SCPCH found following searches of the 246 FUNYBASE proteins on the selected genome sequences (Step 1) was systematically registered in a log file. Similarly, the numbers of candidate SCPCH moved either in the i. “Accepted” or ii. “Rejected – no primers” or iii. “Rejected” folders (see the criteria previously described in Step 3) were also automatically registered in the same file. Finally, all these different results were compared relatively to the number of genomic resources considered in the different PHYLORPH runs.

### Experimental validation

The accuracy of PHYLORPH to find polymorphic markers was tested with a set of four groups of non-model fungi representing 4 different orders among Ascomycota and Basidiomycota. We tested the efficiency of the programme to successfully provide candidate SCPCH that could be readily amplified and sequenced and that showed substantial informative variation across i. two cryptic ITS lineages related to *Erysiphe alphitoides* and *E. quercicola* and included within the oak powdery mildew complex [73], [74], ii. the North American and Eurasian lineages of the root- and heart-rot forest pathogen *Heterobasidion annosum* [75], iii. individuals of *Armillaria ostoyae* causing a root rot disease on Pines [76] and, iv. cryptic species of the *Hymenoscyphus albidus*/*H. pseudoalbidus* (anamorph *Chalara fraxinea*) complex, responsible of an ash decline in Europe [66], [67].

#### PHYLORPH runs

For oak powdery mildews, we requested PHYLORPH to search the complete genomes sequences of *Blumeria graminis* and *Botrytis cinerea* for SCPCH homologous to the 246 protein sequences of *Sclerotinia sclerotiorum* i.e. the closest model species to *Erysiphe* spp. available in FUNYBASE (divergence time about 300 Million of years [My] between Erysiphales (*Erysiphe* spp. and *B. graminis*) and Helotiales [*S. sclerotiorum* and *B. cinerea*]; [77]). Similarly, the 246 protein sequences of *Phanerochaete chrysosporium* were used to search for homologues in the full genome sequences of *Coprinopsis cinereus*, *Laccaria bicolor*, *Pleurotus ostreatus, Postia placenta* and *Schizophyllum commune,* to propose a set of candidate SCPCH for *H. annosum.* This request had been made few months before the first public release of the full genome sequence of this fungus in June 2009 (see at http://genome.jgi-psf.org/Hetan1/Hetan1.info.html). The same six genomic resources were also used to run a search for *A. ostoyae* (divergence time about 230 to 240 My between Polyporales [*P. chrysosporium* and *P. placenta*] and Agaricales [others, including *A. ostoyae* and *H. annosum*]; [78]). Finally, to find SCPCH candidates for the *H. albidus*/*H. pseudoalbidus* complex, we used the single copy genes set from *S. sclerotiorum* and the genomic sequences of *B. graminis*, *B. cinerea*, *Magnaporthe grisea*, *Neurospora crassa* and *Fusarium graminearum* (divergence time about 350–500 My between Leotiomycetes [*Hymenoscyphus* spp., *S. sclerotiorum*, *B. cinerea* and *B. graminis*] and Sordariomycetes [79]). Following each PHYLORPH run, 10 to 14 SCPCH retained into the “Accepted” folder (see Step 3) were selected for primer designs (one or two pairs) based on the topological scores available from the FUNYBASE database. In an attempt to maximize polymorphism levels for *A. ostoyae* and *H. annosum*, we preferentially targeted SCPCH which included primer regions flanking the predicted locations of introns. PHYLORPH indeed provides an automatic prediction of intron/exons boundaries for each SCPCH when the spliced transcript sequence issued from the protein sequence is aligned to its closest homolog from the full genome sequences.

#### Strains tested and DNA extractions

Twenty *E. alphitoides*/*E. quercicola* strains (10/10) were obtained from infected leaves collected in two oak regenerations (mainly *Q. robur*/*Q. petraea*) in France. Total DNA was extracted and diluted following the CTAB protocol described in [73]. Each strain was identified as related to the *E. alphitoides* or the *E. quercicola* lineage by sequencing the internal transcribed spacers (ITS) region of the nuclear ribosomal repeat unit [73]. For *H. albidus*/*H. pseudoalbidus* we used DNA extracted from 30 isolates collected in France described in [67]. DNA from eighteen *H. annosum* isolates collected on *Pinus* spp. for the European (15 isolates collected in France) and North-American types (one isolate from Italy) [75] was extracted using the protocol of [80]. For *A. ostoyae*, we extracted DNA as described in [76] from four isolates collected in four *Pinus sylvestris* populations from the south-west region in France.

#### PCR and sequencing assays

The primer pairs designed for oak powdery mildews and for *A. ostoyae* and *H. annosum* were tested with the following PCR thermal cycling protocols: initial denaturation of 94°C for 3 min, followed by 40 cycles of denaturation at 94°C (45 s), annealing at 55°C (45 s), extension at 72°C (1 min 10 s), and a final extension of 72°C (7 min). If necessary (i.e. when multiband profiles or no PCR product were obtained), PCR cycling was further optimized by assessing a 55-50°C touchdown (45 s) for 40 cycles. In both cases, PCR reactions (1x PCR buffer, 2 mM of MgCl_2,_ 0.8 µM total dNTPs, 0.2 µM primers, 0.75U of Taq Polymerase [Sigma-Aldrich]) were carried out in a 20 µl volume. Amplified products were separated on 2% agarose gels and visualized by ethidium bromide staining. Finally, if the PCR assays yielded discrete bands of the appropriate size, PCR products were excised and cleaned using Qiaquick columns (QIAGen). Experimental validations of the candidate genes for *H. albidus*/*H. pseudoalbidus* were carried out with the same conditions that those described above, except that two different PCR cycles were used: i. a 55–50°C touchdown identical to the one previously described above and ii. an initial denaturation at 94°C for 3 min followed by 35 cycles at 95°C (30 s), a gradient at 50–60°C (30 s), 72°C (1 min 10 s), and a final extension at 72°C (7 min). In all cases, sequencing was then performed using dye labelled terminators (ABI PRISM^TM^ BigDye^TM^ Terminator Cycle Sequencing Ready Reaction Kit) and run on an ABI 3730 automated DNA sequencer. Sequences were edited and manually aligned using BioEdit v.7.0.5 (Hall 1999).

In addition, the amplification efficiencies obtained for the candidates SCPCH tested on oak powdery mildews were compared with those obtained for several genes commonly used to construct fungal phylogenies. The universal primer pairs (listed in Table 2) previously designed for the amplification of the Elongation factor EF-1α, Calmodulin, Chitin synthase I, γ-actin, Histone-3 and -4 and β-tubulin genes [10], [11], [81] were tested on the *E. alphitoides*/*E. quercicola* strains using the “classical” PCR protocol described above (40 cycles with annealing temperature at 55°C).

#### Phylogenetic performance of SCPCH

In order to determine if the SCPCH obtained also performed well in phylogenetic analyses at low evolutionary levels, we used an enlarged taxa sampling for the oak powdery mildews and the *H. albidus*/*H. pseudoalbidus* groups. The SCPCH previously obtained were amplified and sequenced in seven additional related species (*Erysiphe convolvuli*, *Erysiphe cruciferarum*, *Erysiphe trifolii*, *Erysiphe elevata*, *Erysiphe platani* and *Oïdium neolycopersici*) and 4 species (*Ceratocystis platani*, *Ceratocystis fructigena*, *Ceratocystis paradoxa* and *Grosmania clavigera*) for the oak powdery mildew and the *H. albidus*/*H. pseudoalbidus* groups, respectively. The ortholog sequence of *M. grisea* was obtained from the full genome sequence (NCBI Reference Sequence: NZ_AACU00000000.2) and used to root the trees reconstructed for *Hymenoscyphus* spp. and related species. For each taxa set, we used the PCR and sequencing protocols described above. Maximum parsimony (MP) and maximum likelihood (ML) trees were reconstructed among each genes using PAUP ver. 4.0b10 (Swofford 2003). All nucleotide sites were included in the analyses but gaps were treated as missing characters. MP trees were identified by heuristic searches and 100 random additions of sequences (RAS). Confidence was examined using bootstrapping (BS) with 1000 replicates and the heuristic option with 100 RAS per pseudo replicate. Heuristic ML searches were performed with 100 replicates of random sequence addition and tree bisection reconnection (TBR) branch swapping. The best-fit model of sequence evolution for each gene was determined using the Akaike information criterion (AIC) implemented in JModelTest 0.1.1 (Posada 2008). Branch support was evaluated using 100 bootstrap replicates and 100 RAS per pseudo replicate. In MP and ML analyses, monophylies supported by both BS ≥60% were considered as significant. ML combined analyses (e.g. different gene datasets concatenated) were executed through the metapopulation genetic algorithm implemented in MetaPIGA version 2.0 [82] using a mixed-model approach with each search replicated 10,000 times. Topological incongruences detected between trees were statistically evaluated by using the Wilcoxon sign-ranks test under MP and the Shimodaira-Hasegawa multiple comparison test in ML (SH-test; [83] implemented in PAUP, as described in [84].

## Supporting Information

Figure S1 **Distribution of the SCPCH found among the 40 PHYLORPH runs performed for the computational testing.** For example, five SCPCH (FUNYBASE ID FG1010, FG543, FG635, FG646 and FG909) are systematically found in 39 out of the 40 searches made.
(TIF)

Figure S2 **Cross comparison of the three protein datasets (FUNYBASE, PHYLOME-T60 and OrthoMCL-DB) used as the initial step of PHYLORPH.** (A) Number of orthologs shared between two or three databases (showed at the intersections of the Venn diagram) or exclusive to one database (into the circles); (B) Number of fungal species exclusive to one database or shared between two or three databases.
(TIF)

Table S1 **Fungal protein and genome sources used in PHYLORPH.**
(DOC)

Table S2 **List of the 40 different PHYLORPH runs performed for the computational testing.** Those destined to the experimental validation are indicated in bold.
(DOC)
