# Supplementary figures and images for: Finding Single Copy Genes Out of Sequenced Genomes for Multilocus Phylogenetics in Non-Model Fungi
Source: PLoS One. 2011 Apr 13;6(4):e18803. doi: 10.1371/journal.pone.0018803 (PMC3076447; doi:10.1371/journal.pone.0018803)

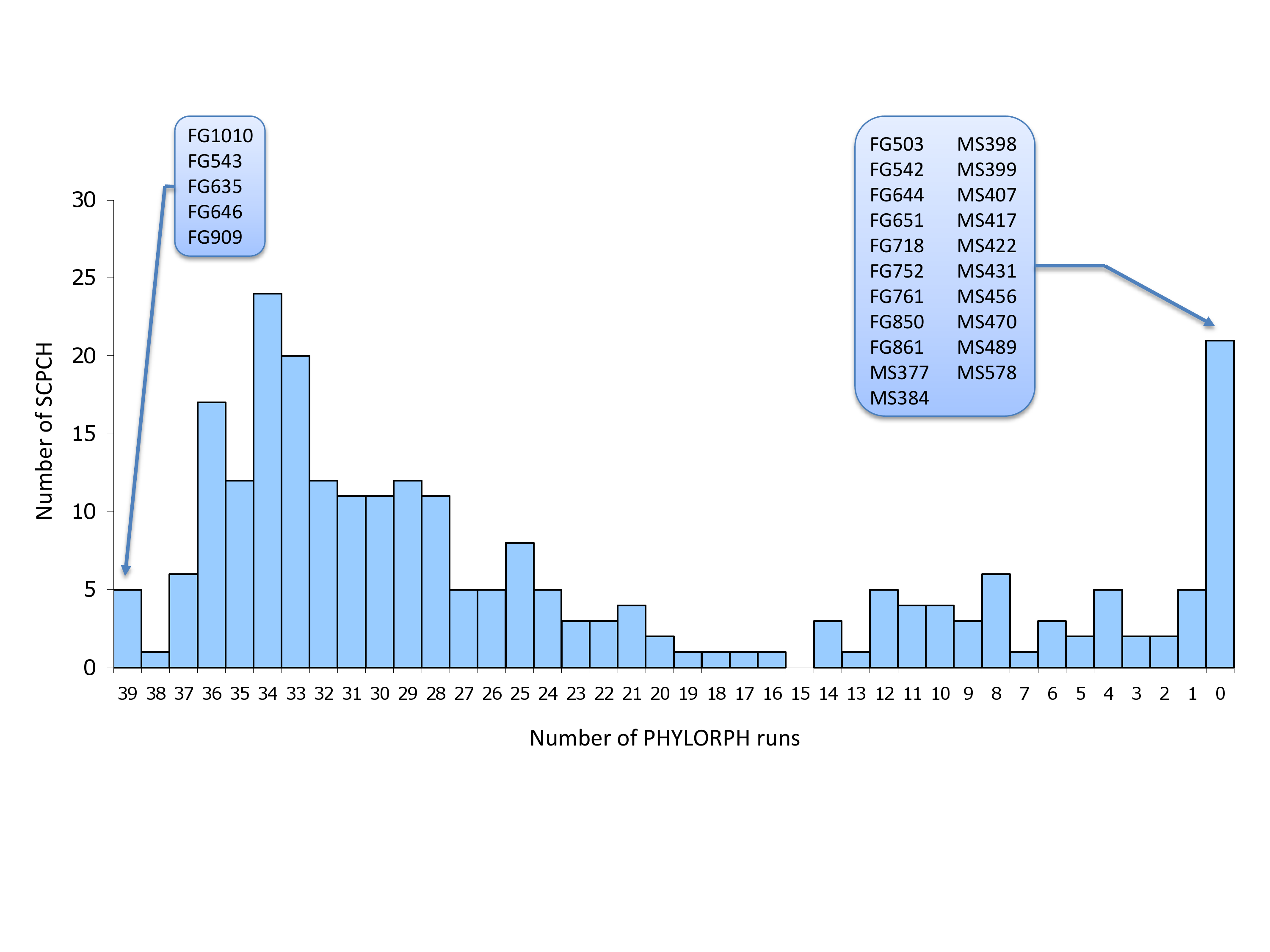

Supplement: Figure S1 — Distribution of the SCPCH found among the 40 PHYLORPH runs performed for the computational testing. For example, five SCPCH (FUNYBASE ID FG1010, FG543, FG635, FG646 and FG909) are systematically found in 39 out of the 40 searches made. (TIF) [file pone.0018803.s001.tif]

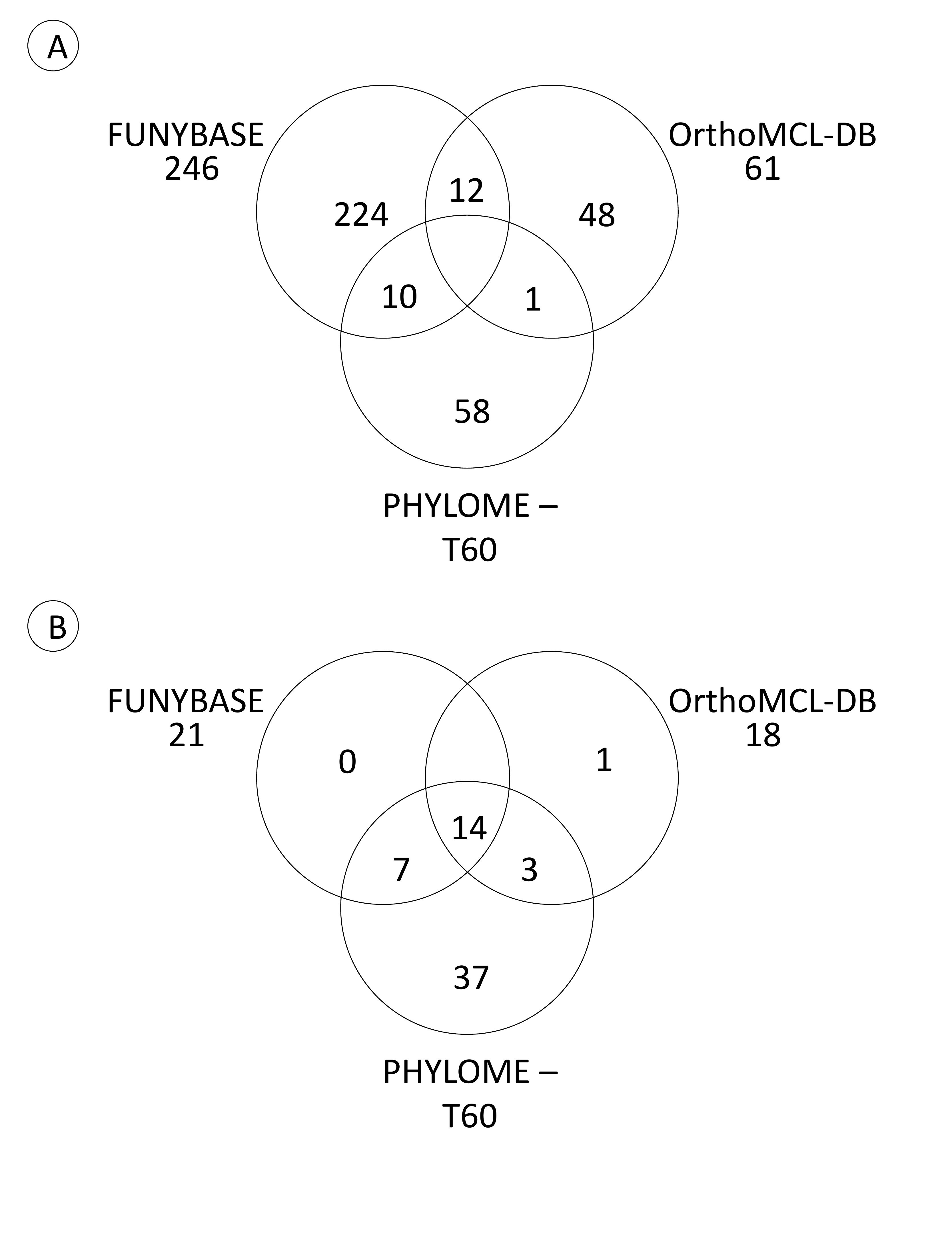

Supplement: Figure S2 — Cross comparison of the three protein datasets (FUNYBASE, PHYLOME-T60 and OrthoMCL-DB) used as the initial step of PHYLORPH. (A) Number of orthologs shared between two or three databases (showed at the intersections of the Venn diagram) or exclusive to one database (into the circles); (B) Number of fungal species exclusive to one database or shared between two or three databases. (TIF) [file pone.0018803.s002.tif]
