# Supplementary material for: Finding Single Copy Genes Out of Sequenced Genomes for Multilocus Phylogenetics in Non-Model Fungi
Source: PLoS One. 2011 Apr 13;6(4):e18803. doi: 10.1371/journal.pone.0018803 (PMC3076447; doi:10.1371/journal.pone.0018803)
Supplement: Table S1 — Fungal protein and genome sources used in PHYLORPH. (DOC) [file pone.0018803.s003.doc]

| Taxonomy^1^ | | | | | | | | | | | | Size (Mb.) | Protein sets^2^ | Genome sources^3^ | |
| --- | --- | --- | --- | --- | --- | --- | --- | --- | --- | --- | --- | --- | --- | --- | --- |
| Phylum | Subphylum | Class | | | Order | | Species | | | | |  |  |  |  |
| Chytridiomycota | | | | | | | | | | | | | | | |
|  | | | | Chytridiomycetes | | | | | | | | | | | |
|  | | | | | | Rhizophydiales | | | | | | | | | |
|  | | | | | | | | | | *Batrachochytrium dendrobatidis* | | 23.7 | P | | Broad |
| Mucoromycotina | | | | | | | | | | | | | | | |
|  | | | | | | Mucorales | | | | | | | | | |
|  | | | | | | | | | | *Phycomyces blakesleeanus* | | 53.9 | P | | JGI |
|  |  |  |  |  |  |  |  |  |  | *Rhizopus oryzae* | | 46.1 | P | | Broad |
| Ascomycota | | | | | | | | | | | | | | | |
|  | Taphrinomycotina | | | | | | | | | | | | | | |
|  | | Schizosaccharomycetes | | | | | | | | | | | | | |
|  | | | | | Schizosaccharomycetales | | | | | | | | | | |
|  | | | | | | | | | | | *Schizosaccharomyces japonicus* | 11.3 | P | Broad | |
|  |  |  |  |  |  |  |  |  |  |  | *Schizosaccharomyces octosporus* | 11.2 |  | Broad | |
|  |  |  |  |  |  |  |  |  |  |  | *Schizosaccharomyces pombe* | 11.5 | F, P, O | Broad | |
|  | | Pneumocystidomycetes | | | | | | | | | | | | | |
|  | | | | | Pneumocystidales | | | | | | | | | | |
|  | | | | | | | | *Pneumocystis carinii* | | | | 8.0 | P | NCBI | |
|  | Pezizomycotina | | | | | | | | | | | | | | |
|  | | Eurotiomycetes | | | | | | | | | | | | | |
|  | | | | | | Eurotiales | | | | | | | | | |
|  | | | | | | | | | | | *Aspergillus clavatus* | 27.9 | P | Broad | |
|  |  |  |  |  |  |  |  |  |  |  | *Aspergillus flavus* | 36.8 | P | Broad | |
|  |  |  |  |  |  |  |  |  |  |  | *Aspergillus fumigatus* | 29.4 | F, P, O | Broad | |
|  |  |  |  |  |  |  |  |  |  |  | *Aspergillus nidulans* | 30.1 | F, P | Broad | |
|  |  |  |  |  |  |  |  |  |  |  | *Aspergillus niger* | 37.2 | P | Broad | |
|  |  |  |  |  |  |  |  |  |  |  | *Aspergillus oryzae* | 37.1 | P, O | Broad | |
|  |  |  |  |  |  |  |  |  |  |  | *Aspergillus terreus* | 29.3 | P | Broad | |
|  |  |  |  |  |  |  |  |  |  |  | *Neosartorya fischeri* | 32.6 | P | Broad | |
|  |  |  |  |  |  |  |  |  |  |  | *Talaromyces stipitatus* | 35.6 |  | NCBI | |
|  | | | | | | Onygenales | | | | | | | | | |
|  | | | | | | | | | | | *Coccidioides immitis* | 28.9 | F, P, O | Broad | |
|  |  |  |  |  |  |  |  |  |  |  | *Coccidioides posadasii* | 28.1 | O | Broad | |
|  |  |  |  |  |  |  |  |  |  |  | *Histoplasma capsulatum* | 33.0 | P | Broad | |
|  |  |  |  |  |  |  |  |  |  |  | *Microsporum canis* | 23.2 |  | Broad | |
|  |  |  |  |  |  |  |  |  |  |  | *Microsporum gypseum* | 23.2 |  | Broad | |
|  |  |  |  |  |  |  |  |  |  |  | *Paracoccidioides brasiliensis* | 32.9 |  | Broad | |
|  |  |  |  |  |  |  |  |  |  |  | *Trichophyton equineum* | 24.1 |  | Broad | |
|  |  |  |  |  |  |  |  |  |  |  | *Trichophyton rubrum* | 22.5 |  | Broad | |
|  |  |  |  |  |  |  |  |  |  |  | *Trichophyton tonsurans* | 23.0 |  | Broad | |
|  |  |  |  |  |  |  |  |  |  |  | *Uncinocarpus reesii* | 22.3 | P | Broad | |
|  | | Leotiomycetes | | | | | | | | | | | | | |
|  | | | | | | Helotiales | | | | | | | | | |
|  | | | | | | | | | | | *Botrytis cinerea* | 38.8 | P | Broad | |
|  |  |  |  |  |  |  |  |  |  |  | *Geomyces destructans* | - |  | Broad | |
|  |  |  |  |  |  |  |  |  |  |  | *Sclerotinia sclerotiorum* | 38.3 | F, P | Broad | |
|  | | | | | | Erysiphales | | | | | | |  | | |
|  | | | | | | | | | | | *Blumeria graminis* | 131.0 |  | NCBI | |
|  | | Sordariomycetes | | | | | | | | | | | | | |
|  | | | | | | Sordariales | | | | | | | | | |
|  | | | | | | | | | | | *Chaetomium globosum* | 34.9 | P | Broad | |
|  |  |  |  |  |  |  |  |  |  |  | *Neurospora crassa* | 41.0 | F, P, O | Broad | |
|  |  |  |  |  |  |  |  |  |  |  | *Neurospora tetrasperma* | 37.8 |  | JGI | |
|  |  |  |  |  |  |  |  |  |  |  | *Podospora anserina* | - | P | IGM | |
|  |  |  |  |  |  |  |  |  |  |  | *Thielavia terrestris* | 36.9 |  | JGI | |
|  |  |  |  |  |  |  |  |  |  |  | *Sordaria macrospora* | 39.8 |  | RUB | |
|  | | | | | | Hypocreales | | | | | | | | | |
|  | | | | | | | | | | | *Fusarium oxysporum* | 61.4 | P | Broad | |
|  |  |  |  |  |  |  |  |  |  |  | *Fusarium verticillioides* | 41.8 | P | Broad | |
|  |  |  |  |  |  |  |  |  |  |  | *Fusarium graminearum* | 36.4 | F, P, O | Broad | |
|  | | | | | | | | | | | *Nectria haematococca* | 51.3 | P | JGI | |
|  |  |  |  |  |  |  |  |  |  |  | *Trichoderma atroviride* | 36.1 |  | JGI | |
|  |  |  |  |  |  |  |  |  |  |  | *Trichoderma reesei* | 33.5 | F, P | JGI | |
|  |  |  |  |  |  |  |  |  |  |  | *Trichoderma virens* | 38.8 |  |  | |
|  | | | | | | Diaporthales | | | | | | |  |  | |
|  | | | | | | | | | | | *Cryphonectria parasitica* | 43.9 |  | JGI | |
|  | | | | | | Ophiostomatales | | | | | | |  | | |
|  | | | | | | | | | | | *Grosmania clavigera* |  |  | NCBI | |
|  | | | | | | *Incertae sedis* | | | | | |  |  |  | |
|  | | | | | | | | | | | *Colletotrichum graminicola* | 50.9 |  | Broad | |
|  |  |  |  |  |  |  |  |  |  |  | *Gaeumannomyces graminis* | 43.62 |  | Broad | |
|  |  |  |  |  |  |  |  |  |  |  | *Magnaporthe grisea* | 41.7 | F, P, O | Broad | |
|  |  |  |  |  |  |  |  |  |  |  | *Magnaporthe oryzae* | 40.8 |  | Broad | |
|  |  |  |  |  |  |  |  |  |  |  | *Magnaporthe poae* | 39.5 |  | Broad | |
|  |  |  |  |  |  |  |  |  |  |  | *Verticillium albo-atrum* | 32.8 |  | Broad | |
|  |  |  |  |  |  |  |  |  |  |  | *Verticillium dahliae* | 32.9 |  | Broad | |
|  | | Dothideomycetes | | | | | | | | | | | | | |
|  | | | | | | Capnodiales | | | | | | | | | |
|  | | | | | | | | | | | *Mycosphaerella fijiensis* | 73.4 | P | JGI | |
|  |  |  |  |  |  |  |  |  |  |  | *Mycosphaerella graminicola* | 39.7 |  | JGI | |
|  |  |  |  |  |  |  |  |  |  |  | *Mycosphaerella pini* | 30.2 |  | JGI | |
|  |  |  |  |  |  |  |  |  |  |  | *Mycosphaerella populorum* | 29.4 |  | JGI | |
|  | | | | | | Pleosporales | | | | | | |  | | |
|  | | | | | | | | | | | *Alternaria brassicicola* | 32.0 |  | JGI | |
|  |  |  |  |  |  |  |  |  |  |  | *Cochliobolus heterostrophus* | 34.9 |  | JGI | |
|  |  |  |  |  |  |  |  |  |  |  | *Pyrenophora tritici-repentis* | 37.8 |  | Broad | |
|  |  |  |  |  |  |  |  |  |  |  | *Stagonospora nodorum* | 37.1 |  | Broad | |
|  | | | Pezizomycetes | | | | | | | | | | | | |
|  | | | | | | Pezizales | | | | | | | | | |
|  | | | | | | | | | | | *Tuber melanosporum* | 125.0 |  | Genoscope | |
|  | Saccharomycotina | | | | | | | | | | | | | | |
|  | | Saccharomycetes | | | | | | | | | | | | | |
|  | | | | | | Saccharomycetales | | | | | | | | | |
|  | | | | | | | | | | | *Ashbya gossypii* | 9.2 | F, O | AGD | |
|  |  |  |  |  |  |  |  |  |  |  | *Candida albicans* | 14.4 | P | Broad | |
|  |  |  |  |  |  |  |  |  |  |  | *Candida dubliniensis* | - | P | - | |
|  |  |  |  |  |  |  |  |  |  |  | *Candida glabatra* | 12.3 | F, P, O | CGD | |
|  |  |  |  |  |  |  |  |  |  |  | *Candida guilliermondii* | 10.6 | P | Broad | |
|  |  |  |  |  |  |  |  |  |  |  | *Candida tropicalis* | 14.7 | P | Broad | |
|  |  |  |  |  |  |  |  |  |  |  | *Clavispora lusitaniae* | 12.1 | F, P | Broad | |
|  |  |  |  |  |  |  |  |  |  |  | *Debaryomyces hansenii* | 12.2 | F, P, O | Broad | |
|  |  |  |  |  |  |  |  |  |  |  | *Kluyveromyces lactis* | 10.6 | F, P, O | NCBI | |
|  |  |  |  |  |  |  |  |  |  |  | *Kluyveromyces thermotolerans* | 10.4 |  | Geno. | |
|  |  |  |  |  |  |  |  |  |  |  | *Kluyveromyces waltii* | - | P | - | |
|  |  |  |  |  |  |  |  |  |  |  | *Lodderomyces elongisporus* |  | P | Broad | |
|  |  |  |  |  |  |  |  |  |  |  | *Pichia angusta* | 9.0 |  | JGI | |
|  |  |  |  |  |  |  |  |  |  |  | *Pichia stipitis* | 15.4 | P, O | JGI | |
|  |  |  |  |  |  |  |  |  |  |  | *Saccharomyces bayanus* | 11.5 | F, P | Broad | |
|  |  |  |  |  |  |  |  |  |  |  | *Saccharomyces castellii* | - | P | - | |
|  |  |  |  |  |  |  |  |  |  |  | *Saccharomyces cerevisiae* | 11.7 | F, P, O | Broad | |
|  |  |  |  |  |  |  |  |  |  |  | *Saccharomyces kluyveri* | 11.3 | P | Geno. | |
|  |  |  |  |  |  |  |  |  |  |  | *Saccharomyces kudriavzevii* | - | P | - | |
|  |  |  |  |  |  |  |  |  |  |  | *Saccharomyces mikatae* | 11.6 | P | Broad | |
|  |  |  |  |  |  |  |  |  |  |  | *Saccharomyces paradoxus* | 11.9 | F,P | Broad | |
|  |  |  |  |  |  |  |  |  |  |  | *Yarrowia lipolytica* | 20.0 | F, P, O | NCBI | |
|  |  |  |  |  |  |  |  |  |  |  | *Zygosaccharomyces rouxii* | 10.4 |  | Geno. | |
| Basidiomycota | | | | | | | | | | | | | | | |
|  | Ustilaginomycotina | | | | | | | | | | | | | | |
|  | | Ustilaginomycetes | | | | | | | | | | | | | |
|  | | | | | | Ustilaginales | | | | | | | | | |
|  | | | | | | | | | | | *Ustilago maydis* | 19.7 | F, P | Broad | |
|  | Pucciniomycotina | | | | | | | | | | | | | | |
|  | | Pucciniomycetes | | | | | | | | | | | | | |
|  | | | | | | Pucciniales | | | | | | | | | |
|  | | | | | | | | | | | *Melampsora larici-populina* | 101.1 |  | JGI | |
|  |  |  |  |  |  |  |  |  |  |  | *Puccinia graminis* | 88.6 | P | Broad | |
|  |  |  |  |  |  |  |  |  |  |  | *Puccinia triticina* | 162.9 |  | Broad | |
|  | | Microbotryomycetes | | | | | | | | | | | | | |
|  | | | | | | Microbotryales | | | | | | | | | |
|  | | | | | | | | | *Microbotryum violaceum* | | | 26.1 |  | | Broad |
|  | | | | | | Sporidiobolales | | | | | | |  | | |
|  | | | | | | | | | | | *Sporobolomyces roseus* | 21.2 | P | JGI | |
|  | | | | | | | | | | | *Rhodotorula graminis* | 21.0 |  | JGI | |
|  | Agaricomycotina | | | | | | | | | | | |  | | |
|  | | Tremellomycetes | | | | | | | | | | |  | | |
|  | | | | | | Tremellales | | | | | | |  | | |
|  | | | | | | | | | | | *Cryptococcus neoformans* | 17.2 | F, P, O | Broad | |
|  |  |  |  |  |  |  |  |  |  |  | *Malassezia globosa* | 9.0 |  | NCBI | |
|  |  |  |  |  |  |  |  |  |  |  | *Tremella mesenterica* | 28.6 |  | JGI | |
|  | | Agaricomycetes | | | | | | | | | | |  | | |
|  | | | | | | Polyporales | | | | | | |  | | |
|  | | | | | | | | | | | *Ceriporiopsis subvermispora* | 39.0 |  | JGI | |
|  |  |  |  |  |  |  |  |  |  |  | *Dichomitus squalens* | 42.8 |  | JGI | |
|  |  |  |  |  |  |  |  |  |  |  | *Fomitopsis pinicola* | 46.3 |  | JGI | |
|  |  |  |  |  |  |  |  |  |  |  | *Phanerochaete chrysosporium* | 35.1 | F, P, O | JGI | |
|  |  |  |  |  |  |  |  |  |  |  | *Postia placenta* | 90.9 | P | JGI | |
|  |  |  |  |  |  |  |  |  |  |  | *Sporotrichum termophile* | 38.7 |  | JGI | |
|  |  |  |  |  |  |  |  |  |  |  | *Trametes versicolor* | 44.8 |  | JGI | |
|  |  |  |  |  |  |  |  |  |  |  | *Wolfiporia cocos* | 50.5 |  | JGI | |
|  | | | | | | Gloeophyllales | | | | | | | | | |
|  | | | | | |  | | | | | *Gloeophyllum trabeum* | 37.2 |  | JGI | |
|  | | | | | | Agaricales | | | | | | |  | | |
|  | | | | | | | | | | | *Agaricus bisporus* var*. burnettii* | 30.2 |  | JGI | |
|  |  |  |  |  |  |  |  |  |  |  | *Agaricus bisporus* var*. bisporus* | 30.6 |  | JGI | |
|  |  |  |  |  |  |  |  |  |  |  | *Coprinus cinereus* | 36.3 | P | Broad | |
|  |  |  |  |  |  |  |  |  |  |  | *Laccaria bicolor* | 64.9 | P, O | JGI | |
|  |  |  |  |  |  |  |  |  |  |  | *Pleurotus ostreatus* | 34.3 |  | JGI | |
|  |  |  |  |  |  |  |  |  |  |  | *Schizophyllum commune* | 38.5 |  | JGI | |
|  | | | | | | Russulales | | | | | |  |  |  | |
|  | | | | | | | | | | | *Heterobasidion annosum* | 33.7 |  | JGI | |
|  | | | | | | Boletales | | | | | | |  | | |
|  | | | | | | | | | | | *Serpula lacrymans* | 42.8 |  | JGI | |
|  | *Incertae sedis* | | | | | | | | | | | | | | |
|  | | | Wallemiomycetes | | | | | | | | | | | | |
|  | | | | | | Wallemiales | | | | | | | | | |
|  |  |  |  |  |  |  | | | | | *Wallemia sebi* | 9.82 |  | JGI | |

^1^Taxonomy based on Kirk et al. [46].

^2^Protein sets available into the F, FUNYBASE (<http://genome.jouy.inra.fr/funybase/>); P, Yeast PHYLOME-T60 (<http://phylomedb.org/phylome_3>) and O, OrthoMCL-DB (<http://orthomcl.org/>) databases.

^3^Genome databases : Broad, Broad institute <http://www.broadinstitute.org/science/data>; JGI, Join Genome institute <http://www.candidagenome.org/>; NCBI, National centre for biotechnology information <http://www.ncbi.nlm.nih.gov/>; ADB, Ashbya genome db <http://agd.vital-it.ch/index.html>; CDB, Candida genome db <http://www.candidagenome.org/>; Genoscope, Centre national de sequençage Genoscope <http://www.genoscope.cns.fr/spip/>; RUB, Rhur-University Bochum <http://c4-1-8.serverhosting.rub.de/public/>; Geno., Génolevure, <http://www.genolevures.org/>; IGM, Institut de Génétique et Microbiologie, Université Paris Sud, <http://podospora.igmors.u-psud.fr/index.php>
