# Supplementary material for: Finding Single Copy Genes Out of Sequenced Genomes for Multilocus Phylogenetics in Non-Model Fungi
Source: PLoS One. 2011 Apr 13;6(4):e18803. doi: 10.1371/journal.pone.0018803 (PMC3076447; doi:10.1371/journal.pone.0018803)
Supplement: Table S2 — List of the 40 different PHYLORPH runs performed for the computational testing. Those destined to the experimental validation are indicated in bold. (DOC) [file pone.0018803.s004.doc]

| No. of genomic resources | FUNYBASE set | Full genome sequences interrogated | Size (Mb) | Total computing time (seconds) | No. of “Accepted” SCPCH |
| --- | --- | --- | --- | --- | --- |
| 3 | *Phanerochaete chrysosporium* | *Coprinopsis cinereus, Pleurotus ostreatus* | 84.4 | 1662 | 169 |
|  | *Trichoderma reesei* | *Chaetomium globosum, Neurospora crassa* | 90.1 | 1499 | 153 |
|  | *Fusarium graminearum* | *Nectria haematococca, Cryphonectria parasitica* | 113.0 | 1816 | 150 |
|  | *Sclerotinia sclerotiorum* | *Botrytis cinerea, Nectria haematococca* | 105.9 | 1500 | 85 |
|  | ***Sclerotinia sclerotiorum*** | ***Botrytis cinerea, Blumeria graminis*** | **215.9** | **1925** | **133** |
|  | *Fusarium graminearum* | *Fusarium verticillioides, Nectria haematococca* | 110.9 | 1632 | 140 |
|  | *Trichoderma reesei* | *Mycosphaerella fijiensis, Alternaria brassicicola* | 119.5 | 1718 | 137 |
|  | *Aspergillus nidulans* | *Coccidioides posadasii, Histoplasma capsulatum* | 76.6 | 1761 | 164 |
|  | *Ustilago maydis* | *Sporobolomyces roseus, Tremella mesenterica* | 61.8 | 1124 | 99 |
| 4 | *Coccidioides immitis* | *Aspergillus oryzae, Coccidioides posadasii, Trichophyton equinum* | 102.4 | 1595 | 148 |
|  | *Ustilago maydis* | *Puccinia graminis, Melampsora larici-populina, Puccinia triticina* | 201.8 | 3759 | 68 |
|  | *Phanerochaete chrysosporium* | *Coprinopsis cinereus, Pleurotus ostreatus, Laccaria bicolor* | 149.2 | 1899 | 135 |
|  | *Fusarium graminearum* | *Trichoderma virens, Colletotrichum graminicola, Verticillium dahliae* | 140.4 | 1912 | 138 |
|  | *Saccharomyces paradoxus* | *Candida lusitaniae, Saccharomyces mikatae, Pichia stipitis* | 48.3 | 1099 | 130 |
|  | *Cryptococcus neoformans* | *Tremella mesenterica, Heterobasidion annosum, Pleurotus ostreatus* | 107.4 | 1922 | 104 |
|  | *Neurospora crassa* | *Botrytis cinerea, Chaetomium globosum, Neurospora tetrasperma* | 126.4 | 1795 | 125 |
|  | *Aspergillus nidulans* | *Aspergillus niger, Aspergillus clavatus, Microsporum gypseum* | 103.8 | 1641 | 147 |
|  | *Cryptococcus neoformans* | *Tremella mesenterica, Heterobasidion annosum, Agaricus bisporus* | 123.5 | 2027 | 97 |
|  | *Cryptococcus neoformans* | *Tremella mesenterica, Heterobasidion annosum, Postia placenta* | 164.0 | 1471 | 34 |
|  | *Saccharomyces paradoxus* | *Mycosphaerella fijiensis, Alternaria brassicicola, Candida guilliermondii* | 125.2 | 1826 | 56 |
|  | *Candida glabrata* | *Candida guilliermondii, Candida tropicalis, Saccharomyces bayanus* | 45.7 | 1084 | 125 |
| 5 | *Magnaporthe grisea* | *Neurospora tetrasperma, Trichoderma virens, Fusarium verticillioides, Verticillium albo-atrum* | 134.3 | 2794 | 120 |
|  | *Coccidioides immitis* | *Aspergillus flavus, Aspergillus clavatus, Microsporum canis, Uncinocarpus reesii* | 123.2 | 1894 | 131 |
|  | *Magnaporthe grisea* | *Mycosphaerella fijiensis, Alternaria brassicicola, Pyrenophora tritici-repentis, Cochliobolus heterostrophus* | 194.0 | 2766 | 117 |
|  | *Ustilago maydis* | *Tremella mesenterica, Heterobasidion annosum, Agaricus bisporus, Serpula lacrymans* | 147.4 | 2687 | 61 |
|  | *Trichoderma reesei* | *Mycosphaerella graminicola, Mycosphaerella fijiensis, Alternaria brassicicola, Cochliobolus heterostrophus* | 194.1 | 2625 | 112 |
|  | *Ustilago maydis* | *Puccinia graminis, Puccinia triticina, Melampsora larici-populina, Sporobolomyces roseus* | 385.9 | 3898 | 40 |
|  | *Phanerochaete chrysosporium* | *Cryptococcus neoformans, Schizophyllum commune, Agaricus bisporus, Agaricus bisporus var burnettii* | 132.2 | 2653 | 109 |
|  | *Candida glabrata* | *Candida guilliermondii, Candida lusitaniae, Saccharomyces mikatae, Saccharomyces bayanus* | 54.7 | 1419 | 99 |
|  | *Fusarium graminearum* | *Trichoderma atroviride, Nectria haematococca, Sporotrichum termophile, Verticillium albo-atrum* | 176.8 | 3030 | 118 |
| 6 | *Ustilago maydis* | *Cryptococcus neoformans, Puccinia graminis, Puccinia triticina, Melampsora larici-populina, Sporobolomyces roseus* | 403.1 | 4362 | 26 |
|  | *Neurospora crassa* | *Trichoderma atroviride, Fusarium verticillioides, Nectria haematococca, Sporotrichum termophile, Cryphonectria parasitica* | 226.7 | 3468 | 125 |
|  | *Aspergillus nidulans* | *Aspergillus niger, Aspergillus terreus, Aspergillus clavatus, Microsporum canis, Trichophyton rubrum* | 155.6 | 2427 | 131 |
|  | ***Sclerotinia sclerotiorum*** | ***Botrytis cinerea, Blumeria graminis, Fusarium graminearum, Neurospora crassa, Magnaporthe grisea*** | **335.1** | **3331** | **76** |
|  | ***Phanerochaete chrysosporium*** | ***Postia placenta, Coprinopsis cinereus, Pleurotus ostreatus, Laccaria bicolor, Schizophyllum commune*** | **278.6** | **2093** | **31** |
|  | *Magnaporthe grisea* | *Sporotrichum termophile, Cryphonectria parasitica, Colletotrichum graminicola, Verticillium dahliae, Verticillium albo-atrum* | 215.2 | 2763 | 120 |
|  | *Coccidioides immitis* | *Aspergillus fumigatus, Microsporum gypseum, Trichophyton tonsurans, Uncinocarpus reesii, Coccidioides posadasii* | 139.0 | 2225 | 118 |
|  | *Coccidioides immitis* | *Aspergillus fumigatus, Trichophyton tonsurans, Aspergillus niger, Histoplasma capsulatum, Paracoccidioides brasiliensis* | 168.4 | 2360 | 116 |
|  | *Cryptococcus neoformans* | *Phanerochaete chrysosporium, Coprinopsis cinereus, Schizophyllum commune, Agaricus bisporus, Serpula lacrymans* | 193.7 | 3518 | 72 |
|  | *Phanerochaete chrysosporium* | *Coprinopsis cinereus, Schizophyllum commune, Agaricus bisporus, Serpula lacrymans, Pleurotus ostreatus,* | 195.9 | 3204 | 104 |
